# Insight Into the Properties and Immunoregulatory Effect of Extracellular Vesicles Produced by *Candida glabrata*, *Candida parapsilosis*, and *Candida tropicalis* Biofilms

**DOI:** 10.3389/fcimb.2022.879237

**Published:** 2022-06-06

**Authors:** Kamila Kulig, Elzbieta Karnas, Olga Woznicka, Patryk Kuleta, Ewa Zuba-Surma, Elzbieta Pyza, Artur Osyczka, Andrzej Kozik, Maria Rapala-Kozik, Justyna Karkowska-Kuleta

**Affiliations:** ^1^ Department of Analytical Biochemistry, Faculty of Biochemistry, Biophysics and Biotechnology, Jagiellonian University, Kraków, Poland; ^2^ Department of Cell Biology, Faculty of Biochemistry, Biophysics and Biotechnology, Jagiellonian University, Kraków, Poland; ^3^ Department of Cell Biology and Imaging, Institute of Zoology and Biomedical Research, Jagiellonian University, Kraków, Poland; ^4^ Department of Molecular Biophysics, Faculty of Biochemistry, Biophysics and Biotechnology, Jagiellonian University, Kraków, Poland; ^5^ Department of Comparative Biochemistry and Bioanalytics, Faculty of Biochemistry, Biophysics and Biotechnology, Jagiellonian University, Kraków, Poland

**Keywords:** extracellular vesicles, cytokines, pathogenic fungi, candidiasis, biofilm

## Abstract

Currently, non-*albicans Candida* species, including *C. tropicalis*, *C. glabrata*, and *C. parapsilosis*, are becoming an increasing epidemiological threat, predominantly due to the distinct collection of virulence mechanisms, as well as emerging resistance to antifungal drugs typically used in the treatment of candidiasis. They can produce biofilms that release extracellular vesicles (EVs), which are nanometric spherical structures surrounded by a lipid bilayer, transporting diversified biologically active cargo, that may be involved in intercellular communication, biofilm matrix production, and interaction with the host. In this work, we characterize the size and protein composition of these structures for three species of non-*albicans Candida* fungi forming biofilm, indicating considerable heterogeneity of the investigated population of fungal EVs. Examination of the influence of EVs on cytokine production by the human monocytic cell line THP-1 differentiated into macrophage-like cells revealed that the tested vesicles have a stimulating effect on the secretion of tumor necrosis factor α and interleukin 8, while they reduce the production of interleukin 10. This may indicate the proinflammatory nature of the effect of EVs produced by these species on the host immune cells. Moreover, it has been indicated that vesicles may be involved in *C. tropicalis* biofilm resistance to fluconazole and caspofungin. This reveals the important role of EVs not only in the physiology of *C. tropicalis*, *C. glabrata*, and *C. parapsilosis* fungi but also in the pathogenesis of infections associated with the production of fungal biofilm.

## Introduction

Living in a biofilm is a natural and universal form of existence for a broad variety of microorganisms capable of infecting the human host. Bacterial and fungal pathogens that form multicellular communities might gain protection against unfavorable environmental conditions, applied antimicrobial therapies, and the host’s defense mechanisms (Nobile and Johnson, 2015). Microbial consortia also incessantly affect the host immune system and modulate its function and response, often becoming the cause of recurrent and persistent infections difficult to treat (Chandra et al., 2007; Pereira et al., 2021). One of the most widespread fungal pathogens capable of forming biofilm and causing a wide spectrum of different types of infections in humans are the opportunistic pathogenic yeasts from the *Candida* genus, including the most frequently identified species *C. albicans* (Netea et al., 2015; Tsui et al., 2016). However, other *Candida* species, including *C. tropicalis*, *C. glabrata*, and *C. parapsilosis*, are becoming an increasing epidemiological threat, predominantly due to the distinct collection of virulence mechanisms, as well as emerging resistance to antifungal drugs typically used in the treatment of candidiasis (Silva et al., 2012; Arendrup, 2013; Giacobbe et al., 2020; Zhang et al., 2020). Non-*albicans Candida* (NAC) species also possess the ability to produce biofilm; however, a considerable species- and strain-specific variety of biofilm production capacity for *C. tropicalis*, *C. glabrata*, and *C. parapsilosis* was observed, reflected in the differences in biofilm architecture, complexity, and composition of the biofilm matrix (Silva et al., 2011; Cavalheiro and Teixeira, 2018). Nevertheless, despite some interspecies diversity, generally, the ability to create a biofilm gives pathogenic *Candida* fungi an advantage in maintaining infection and enhancing virulence potential compared with planktonic cells (Cavalheiro and Teixeira, 2018). The adherence of microbial cells to biotic or abiotic surfaces, their coexistence in the community in high density, and embedding in a composite biofilm-supporting matrix not only might make the biofilm-forming cells different from free-floating ones but also contribute to acquiring novel virulence properties by the complex biofilm structure (Xu et al., 2022). These are mainly related to the increased difficulty in the removal of biofilm-forming pathogens from the inhabited surfaces and to the disturbances in the effective action of antimicrobial drugs (Ponde et al., 2021). In studies recently presented by Sasani et al., in 2021, *C. tropicalis* strains isolated from patients with candidemia and characterized by increased metabolic activity and high biofilm mass were related to higher mortality rates, and biofilms formed by these strains showed increased resistance to azole drugs (Sasani et al., 2021). Moreover, research presented previously by Tumbarello et al. (2007) demonstrated the correlation between the biofilm-forming ability of *C. albicans* and *C. parapsilosis* and the mortality of patients with candidemia caused by these species. Such reports indicate the urgent necessity to study in detail the characteristics of this sedentary form of existence of fungal pathogens, which constitute a real threat to human health and life.

In addition to various well-known mechanisms contributing to fungal virulence, the microbial cells that create biofilm produce also extracellular vesicles (EVs)—nanometric spherical structures surrounded by a lipid bilayer, carrying a diversified biologically active cargo—that are involved in intercellular communication, biofilm matrix production, and interaction with the host (Bleackley et al., 2019; Garcia-Ceron et al., 2021; Piffer et al., 2021). EVs produced by *C. albicans* biofilm differ from those produced by planktonic cells not only in the size and heterogeneity of the population but also in the protein composition. In studies presented by Zarnowski et al. (2018), 34% of the identified EV-related proteins were unique to the biofilm state, and several other shared proteins were also enriched in biofilm-derived EVs compared with vesicles from planktonic cells. Proteins carried by vesicles released by *C. albicans* biofilm might be involved in the production of the biofilm matrix through their enzymatic activity, and EVs might directly provide other matrix-building components like lipids and polysaccharides—glucan and mannan—thus contributing to the resistance of *C. albicans* biofilm to fluconazole by entrapping the antifungal drug within the matrix structure (Taff et al., 2012; Zarnowski et al., 2018; Zhao et al., 2021). Moreover, *C. albicans* biofilm-derived EVs play a role not only in biofilm matrix production and in reducing biofilm susceptibility to antifungals, but they are also involved in other stages of biofilm formation, like fungal cell adhesion and biofilm dispersion (Zarnowski et al., 2021). Hence, they contribute strongly and in multiple ways to the creation of this complex microbial community.

*Candida albicans* EVs have been characterized the most thoroughly so far regarding *Candida* fungi. For other species, the available data are incomplete. A detailed comparison has been recently performed by Zamith-Miranda et al. (2021) between EVs released by *C. albicans* and *C. auris* grown as planktonic cells, and the vesicle content was identified as significantly different for these two *Candida* species, including the varied abundance of 393 vesicular proteins when the two species were compared. Such interspecific variability of vesicles resulted also in diverse effects on the host cells (Zamith-Miranda et al., 2021). In our recent paper, we characterized the EVs produced by free-floating cells of three medically important NAC species—*C. tropicalis*, *C. parapsilosis*, and *C. glabrata*. However, given the possible dissimilarity of the EVs produced by the biofilm form, it is also particularly important to characterize the vesicles produced by the biofilm fungal community, especially that the abovementioned NAC species showing the ability to form complex sessile structures are currently the subject of growing interest and importance. Therefore, in the current research, we aimed to characterize the biofilm-derived EVs of *C. tropicalis*, *C. parapsilosis*, and *C. glabrata* along with the analysis of their immunoregulatory properties.

## Materials and Methods

### Fungal Strains and Culturing Conditions

*Candida glabrata* (Anderson) Meyer et Yarrow strain CBS138 (ATCC^®^ 2001™), *C. tropicalis* (Castellani) Berkhout strain T1 (ATCC^®^ MYA-3404™), and *C. parapsilosis* (Ashford) Langeron et Talice strain CDC 317 (ATCC^®^ MYA-4646™) were purchased from the American Type Culture Collection (Manassas, VA, USA) and cultured at 30°C for 18 h in YPD medium (1% yeast extract, 2% soybean peptone, and 2% glucose, Sigma, St. Louis, MO, USA) on an orbital shaker MaxQ 6000 (170 rpm) (Thermo Fisher Scientific, Waltham, MA, USA). Then, cells were harvested by centrifugation (3,000*g*, 5 min); the cell pellets were washed twice with sterile phosphate-buffered saline (PBS), pH 7.4 (Biowest, Nuaillé, France); and 10^8^ *C. tropicalis* and 10^9^ *C. glabrata* or *C. parapsilosis* cells were inoculated into 100 ml of RPMI 1640 medium (Biowest) and cultured in roller bottles (Corning Inc., New York, NY, USA) at 37°C with roller rack speed 3 rpm for 48 h (with medium exchange after 24 h).

### Extracellular Vesicle Isolation

Medium portions collected after every 24 h of biofilm culture were pooled and EVs were then isolated using a previously described protocol (Karkowska-Kuleta et al., 2020) with minor modifications. Briefly, supernatants from cultures of *C. glabrata*, *C. tropicalis*, and *C. parapsilosis* were centrifuged twice at 4,000*g* for 15 min at 4°C, each time discarding the cell pellet, and then concentrated using an Amicon Ultra-15 Centrifugal Filter Unit with a 100-kDa cutoff (Merck, Darmstadt, Germany) with the addition of cOmplete Protease Inhibitor Cocktail (Roche, Basel, Switzerland). After centrifugation for 5 min at 5,000*g* and discarding the pellet, the concentrated medium was filtered using an Ultrafree-CL Centrifugal Filter with a pore size of 0.65 µm (Merck) and ultracentrifuged in an Optima™ L-90K Ultracentrifuge (Beckman Coulter, Brea, CA, USA) at 4°C for 1 h at a rotor speed of 45,000 rpm, which corresponds to a relative centrifugal field of 184,000*g* (*k* factor 85), using a fixed-angle type 50.2 Ti Rotor and polycarbonate thick wall centrifuge tubes (13 × 64 mm) with 13-mm-diameter Delrin tube adapters. After washing with PBS buffer once, the ultracentrifugation step was repeated and the obtained EVs were transferred in 400 µl of PBS to Eppendorf tubes and stored at −80°C.

### EV Observation and Measurements of Vesicle Size and Concentration

As previously described (Karkowska-Kuleta et al., 2020), to observe EVs, a JEOL JEM-2100 HT transmission electron microscope (JEOL, Tokyo, Japan) and negative stain transmission electron microscopy were used with formvar-coated, 300-mesh copper grids prepared for each EV sample using 2% uranyl acetate (Chemapol, Prague, Czech Republic). The size and concentration of EVs were measured as described in a previous work (Karkowska-Kuleta et al., 2020), using the nanoparticle tracking analysis (NTA) and NanoSight NS300 system with camera type sCMOS, laser Blue488, and NTA software Version 3.4 (Malvern Instruments, Malvern, UK).

### Protein, Phospholipid, and Carbohydrate Concentration Measurements

Protein concentration was measured with *o*-phthalaldehyde (OPA; Sigma) in four biological replicates in samples containing vesicles of each *Candida* species, and the fluorescence intensity was measured with excitation and emission wavelengths of 340 and 455 nm, respectively. The phospholipid concentration in the EV preparations was determined using the Phospholipid Assay Kit MAK122 (Sigma) and the carbohydrate concentration with the Total Carbohydrate Assay Kit MAK104 (Sigma) in three biological replicates for each species, strictly in accordance with the manufacturer’s instructions. Absorbance at 570 nm (MAK122) or 490 nm (MAK104) and fluorescence intensity (OPA) were measured using a Synergy H1 Microplate Reader (BioTek Instruments, Winooski, VT, USA).

### Identification of EV Proteins With Liquid Chromatography-Coupled Tandem Mass Spectrometry

For each tested *Candida* strain, 1 × 10^10^ EVs were suspended in 150 µl of 100 mM Tris–HCl buffer, pH 7.6, with 1% sodium dodecyl sulfate and sonicated in four cycles of 30 s with UP50H Compact Lab Homogenizer (amplitude 80%, cycle 0.5, 50 W, 30 kHz; Hielscher Ultrasonics, Teltow, Germany). Then, the solutions were shaken for 5 min at 95°C and centrifuged for 15 min at 12,000*g*, and after that, the proteins in the supernatant were precipitated by the addition of one volume of trichloroacetic acid to four volumes of the sample. After overnight incubation at −20°C, the samples were centrifuged at 10,000*g* for 15 min at 10°C and washed two times with ice-cold acetone. The obtained pellets were suspended in 100 µl of 10 mM HEPES buffer, pH 8.5. Further processing and analysis of the samples were according to the protocols described by Surman et al. (2019). Briefly, the samples were prepared using paramagnetic bead technology based on the Single-Pot Solid-Phase-Enhanced Sample Preparation (SP3) (Hughes et al., 2014) with the use of SpeedBeads™ GE45152105050250 and GE65152105050250 (Sigma-Aldrich) mixed in an equal ratio, and proteins were reduced with dithiothreitol, alkylated with iodoacetamide, and digested with Trypsin/Lys-C Mix (Promega, Mannheim, Germany). Peptides obtained after tryptic digestion were identified using MS/MS analysis after prior separation on a trap column (Acclaim PepMap 100 C18, 75 μm × 20 mm, 3 μm particle, Thermo Fisher Scientific) and on an analytical column (Acclaim PepMap RSLC C18, 75 µm × 500 mm, 2 µm particle, Thermo Fisher Scientific) with the use of UltiMate 3000 RSLCnano System coupled with Q-Exactive mass spectrometer (Thermo Fisher Scientific) with DPV-550 Digital PicoView nanospray source (New Objective, Woburn, MA, USA). The Q-Exactive mass spectrometer was operated in a data-dependent mode applying a top-eight method. Full-scan MS spectra were obtained with a resolution of 70,000 at *m*/*z* 200 with an automatic gain control (AGC target) of 1 × 10^6^. The MS/MS spectra were obtained with a resolution of 35,000 at *m*/*z* 200 with an AGC target of 3 × 10^6^. The maximum ion accumulation times were 120 and 110 ms for the full MS and MS/MS scans, respectively. Peptides were dynamically excluded from fragmentation within 30 s. The RAW files were processed by the Proteome Discoverer platform (v.1.4, Thermo Fisher Scientific) and searched using a locally installed MASCOT search engine (v.2.5.1, Matrix Science, London, UK). The NCBI database (released April 2022) was used with the following taxonomy restrictions: *C. glabrata* (25,798 sequences), *C. parapsilosis* (8,627 sequences), or *Candida tropicalis* (7,188 sequences). The following parameters were applied: fixed modification—cysteine carbamidomethylation; variable modifications—methionine oxidation; peptide mass tolerance—10 ppm; and fragment mass tolerance—20 mmu. Only tryptic peptides with up to one missed cleavage were considered. The mass spectrometry proteomics data have been deposited to the ProteomeXchange Consortium *via* the PRIDE partner repository (Perez-Riverol et al., 2022) with the dataset identifier PXD033327 (*C. glabrata*), PXD033299 (*C. parapsilosis*), and PXD033300 (*C. tropicalis*).

### Culture of THP-1 Cells

The human monocytic cell line THP-1 (Sigma) was cultured in RPMI 1640 medium (Biowest) supplemented with 10% fetal bovine serum (FBS) (Thermo Fisher Scientific) at 37°C in an atmosphere of 5% CO_2_ and 95% humidity. Differentiation from monocytes to macrophage-like cells was performed by treating cells with 10 ng/ml of phorbol 12-myristate 13-acetate (PMA) added to the medium with 100 U/ml penicillin and 100 mg/ml streptomycin (both from Biowest) for 48 h (with medium exchange after 24 h). After differentiation, the medium was replaced with fresh medium without PMA for 3 h, but with 5% FBS, 100 U/ml penicillin, and 100 mg/ml streptomycin, and then the macrophage-like cells (5 × 10^5^ cells per well of a 24-well microplate) were incubated with EVs of each investigated *Candida* species with a cell-to-EV ratio of 1:100,000 for 24 h, at 37°C. After THP-1 cell stimulation by EVs, the cells were discarded by centrifugation (1,000 rpm, 5 min), and the supernatants were collected for further analysis of cytokine production. For each *Candida* species, three independent experiments were performed.

### ELISA Analysis

The levels of selected cytokines [IL-8, tumor necrosis factor α (TNF-α), IL-10] produced by macrophage-like cells were measured using Human IL-8 ELISA Set, Human TNF ELISA Set, and Human IL-10 ELISA Set (BD OptEIA™) strictly in accordance with the manufacturer’s instructions.

### Biofilm Formation in the Presence of EVs

*Candida tropicalis* 1 × 10^5^ cells were placed in 100 µl of RPMI 1640 medium (Biowest) into the wells of a Corning^®^ 96-well black/clear flat bottom polystyrene high bind microplate (Corning Inc., Corning, NY, USA) for 90 min at 37°C in an atmosphere of 5% CO_2_ and 95% humidity. After removing the medium with non-adherent cells and washing the wells once with 200 µl of PBS buffer, 1 × 10^8^ *C. tropicalis* biofilm-derived EVs in 100 µl of RPMI 1640 medium were added to the adherent cells and further incubated for 90 min under the same conditions. After this time, an additional 100 µl of the medium was added to the wells, and fluconazole or caspofungin (both from Sigma) was introduced into some of the wells to a final concentration of 0.5 or 0.01 µg/ml, respectively. After 24 h of incubation under the same conditions, the biofilms were washed three times with 200 µl of PBS buffer, and optical density was measured at 600 nm with the nine-point area scan reading method. Then, to estimate the biofilm metabolic activity, the XTT (sodium 3′-[1-(phenylaminocarbonyl)-3,4-tetrazolium]-bis (4-methoxy6-nitro) benzene sulfonic acid hydrate) (Thermo Fisher Scientific) test was carried out. For this purpose, 100 µl of RPMI 1640 medium without phenol red (Biowest) was added to the biofilm, and then a 50-µl mixture containing XTT at a final concentration of 1 mg/ml and PMS (*N*-methyl dibenzopyrazine methyl sulfate) at a final concentration of 5 µg/ml (Sigma) was added for 60 min. Independently, cells from each well were mechanically detached and, after thorough mixing, transferred to YPD agar plates for CFU counting after 48 h of incubation at 30°C.

## Results

**Figure 1:**
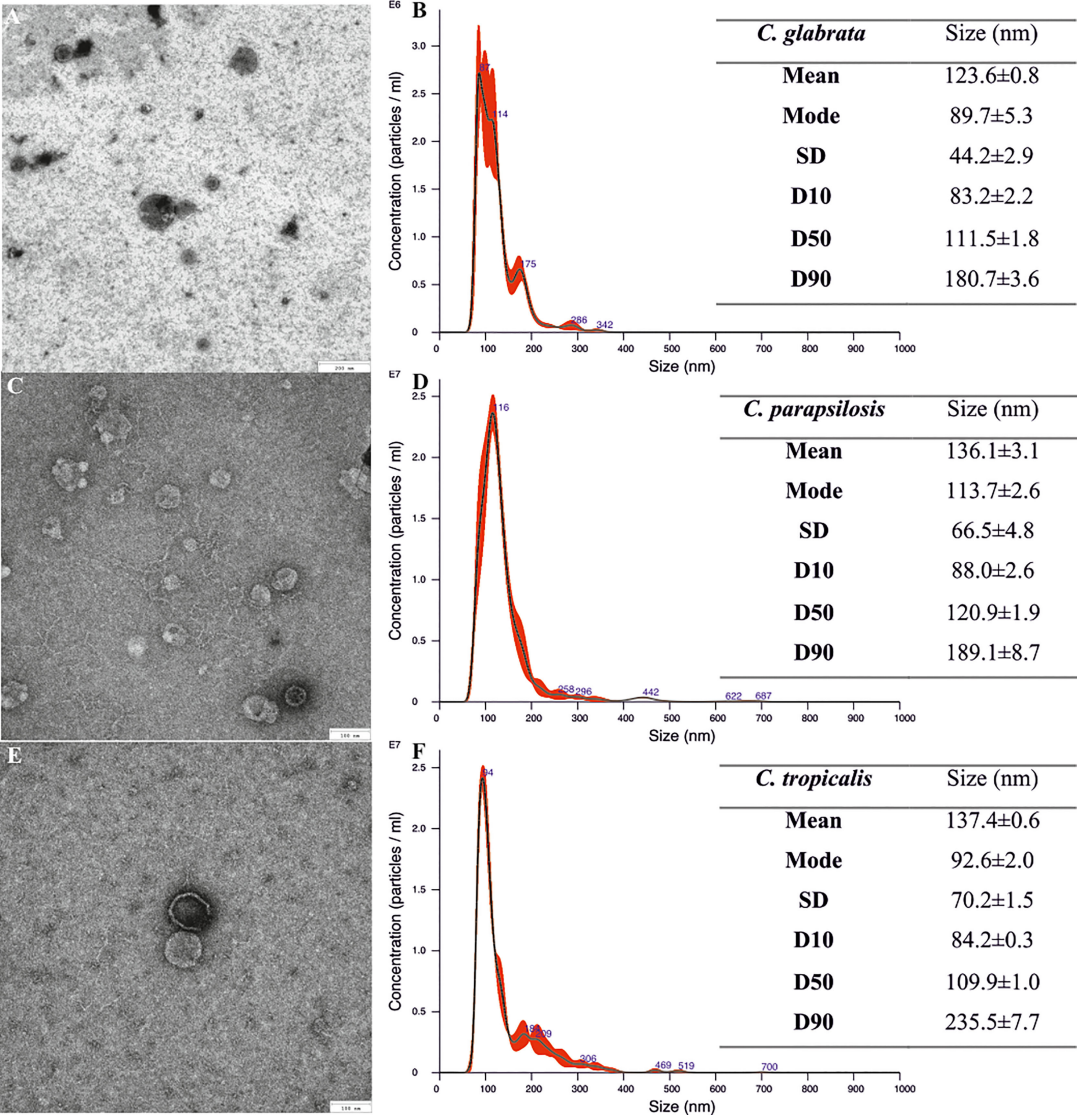
Size characteristics of *Candida glabrata*, *Candida parapsilosis*, and *Candida tropicalis* biofilm-derived extracellular vesicles (EVs). TEM images of EVs **(A, C, E)** and NTA particle size distribution analysis **(B, D, F)**; representative histograms of the average size distribution from three measurements of a single sample (black line). Numbers show the maxima of particular peaks and red areas indicate the standard deviation (SD) between measurements. Tables include EVs’ size parameters measured by NTA; factors D10, D50, and D90 mean that 10%, 50%, and 90% of the EV population had a diameter of less than or equal to the given value. Data are presented as means ± SEM.

The fungal culture initiated by the defined number of *Candida* cells was further maintained in rotating culture bottles in RPMI 1640 medium to generate a complex 48-h biofilm, and then the vesicles were isolated from the concentrated post-culture supernatants. The presence of EVs in the obtained preparations was confirmed by TEM imaging, and the achieved pictures for *C. glabrata* (**Figure 1A**), *C. parapsilosis* (**Figure 1C**), and *C. tropicalis* (**Figure 1E**) indicated the presence of spherical structures of various sizes.

NTA that was performed to determine the number of particles in EV-containing samples showed that biofilms started by 1 × 10^9^ *C. glabrata* cells produced 7.73 ± 0.07 × 10^9^ particles (about 8 per cell) and those by 1 × 10^9^ *C. parapsilosis* cells produced 2.64 ± 0.37 × 10^10^ particles (about 26 per cell), while biofilms initiated by 1 × 10^8^ *C. tropicalis* cells produced 4.36 ± 0.1 × 10^9^ particles (about 44 per cell). The average particle sizes were also measured using NTA and the diameters of EVs produced by *C. glabrata* (**Figure 1B**) and *C. parapsilosis* (**Figure 1D**) ranged from around 80 to 180 nm for 90% of the total particle population, while for *C. tropicalis* (**Figure 1F**), these values were almost up to 245 nm.

The measured average concentrations of proteins, phospholipids, and carbohydrates per 1 × 10^10^ EVs for each *Candida* species are listed in **Table 1**. The highest protein, lipid, and carbohydrate contents were observed in EVs produced by *C. tropicalis* biofilms. The lowest protein and lipid concentrations were identified in EVs from *C. parapsilosis* biofilms, and the lowest average carbohydrate content was indicated for *C. glabrata* biofilm-originated EVs.

**Table 1:** Average protein, lipid, and carbohydrate contents in EVs released by *Candida glabrata*, *Candida parapsilosis*, and *Candida tropicalis* biofilms.

	Protein content (micrograms per 1 × 10^10^ vesicles)	Phospholipid content (nanomoles of lecithin equivalents per 1 × 10^10^ vesicles)	Carbohydrate content (micrograms per 1 × 10^10^ vesicles)
*C. glabrata*	6.88 ± 1.51	9.15 ± 0.29	3.18 ± 0.87
*C. parapsilosis*	4.37 ± 3.57	5.09 ± 1.20	4.66 ± 0.33
*C. tropicalis*	11.04 ± 5.75	17.71 ± 2.42	10.62 ± 2.26

**Figure 2:**
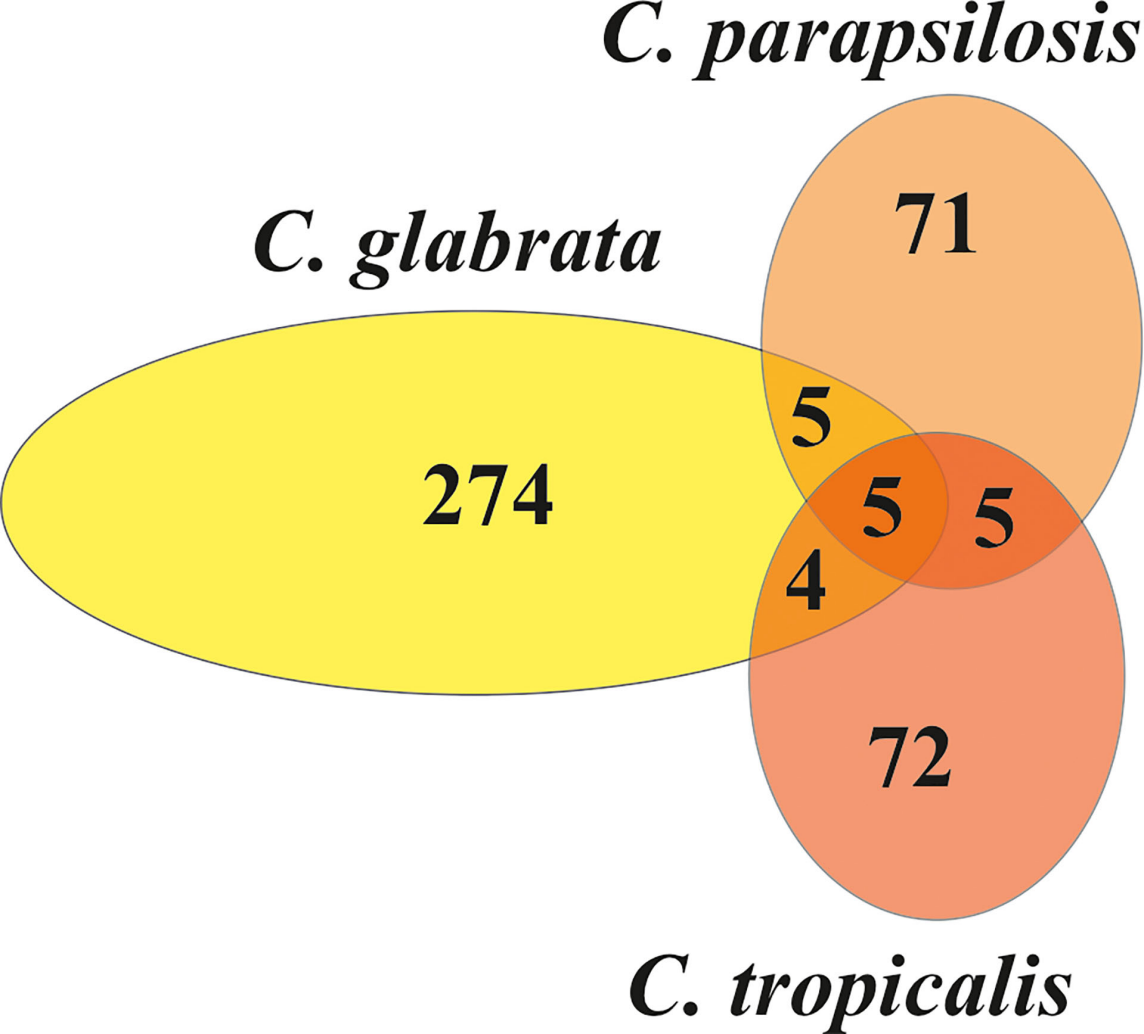
The Venn diagram representing the numbers of individual and common proteins among EVs from *C. glabrata*, *C. parapsilosis*, and *C. tropicalis* biofilms.

Further characterization of fungal EVs included vesicular protein identification using liquid chromatography-coupled tandem mass spectrometry analysis preceded by EV lysis and protein hydrolysis with trypsin. There were 288 proteins identified from *C. glabrata*, 86 from *C. parapsilosis*, and 87 from *C. tropicalis* biofilms (**Supplementary Table 1**). Among the proteins identified for *C. glabrata* EVs, an abundant group of proteins involved in cell wall organization might be distinguished, including among others the cell wall protein Scw11, the cell wall mannoprotein Cwp1, the 1,3-beta-glucan-linked cell wall protein Pir1, the GPI-anchored protein Ecm33, and glycoside hydrolase of the Gas/Phr family Gas1. For EVs from *C. parapsilosis*, this group of proteins was represented by the cell surface mannoprotein Mp65, the GPI-anchored cell wall protein Ecm33, the secreted yeast cell wall protein Ywp1, and 1,3-beta-glucosyltransferase Bgl2. Proteins involved in cell wall organization identified in *C. tropicalis* EVs were the cell surface mannoprotein Mp65, 1,3-beta-glucosidase Bgl2, GPI-anchored cell wall transglycosylase Crh11, and beta-1,3-glucanosyltransferase Pga4. There were some proteins identified in EVs which were shared between species (**Figure 2**). Five proteins were shared among all the tested species: protein similar to alpha agglutinin anchor subunit Tos1, cell wall protein Scw11, protein from the Hsp70 chaperone family, 60S ribosomal protein L12, and glyceraldehyde-3-phosphate dehydrogenase Tdh3. Five proteins—ABC transporter, the GPI-anchored cell wall protein Ecm33, mitochondrial F-ATPase beta subunit Atp2, and two proteins from the SUR7 family—were common to *C. glabrata* and *C. parapsilosis.* Moreover, five proteins—cell surface mannoproteins Mp65, Ywp1, and Bgl2; autophagy-related protein 9 Atg9; and the adhesin-like protein Sim1—were common to *C. parapsilosis* and *C. tropicalis.* Four proteins shared by *C. glabrata* and *C. tropicalis* were enolase, alcohol dehydrogenase, elongation factor 2, and ATP synthase alpha subunit Atp1.

**Figure 3:**
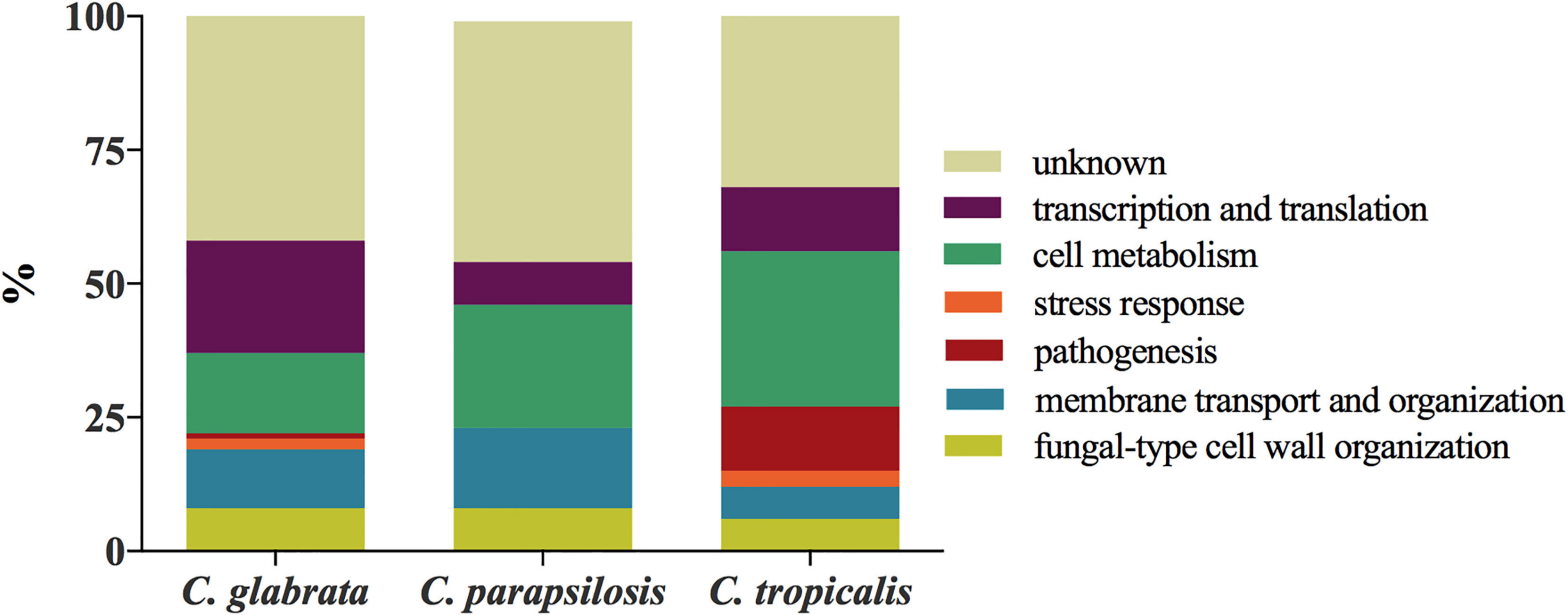
Functional classification of proteins identified for *C. glabrata*, *C. parapsilosis*, and *C. tropicalis* biofilm-originated EVs.

Proteins identified from EVs produced by *C. glabrata*, *C. parapsilosis*, and *C. tropicalis* biofilms were grouped, as previously described (Karkowska-Kuleta et al., 2020), into seven functional classes on the basis of protein descriptions and Gene Ontology (GO) annotations included in the Candida Genome Database (CGD, http://www.candidagenome.org) (Arnaud et al., 2005), UniProtKB Database (https://www.uniprot.org) (UniProt Consortium, 2019), and KEGG (Kyoto Encyclopedia of Genes and Genomes; https://www.genome.jp/kegg/) (Kanehisa and Goto, 2000): i) fungal-type cell wall organization, ii) membrane transport and organization, iii) stress response, iv) pathogenesis, v) cell metabolism, vi) transcription and translation, and vii) proteins of unknown function. Bar charts representing the percentage contents of proteins for each functional group are shown in **Figure 3**. The largest group of proteins found in EVs was transcription and translation (21%) for *C. glabrata* and cell metabolism (22%) for *C. parapsilosis* and *C. tropicalis* (29%). The least represented functional group was pathogenesis (1%) for *C. glabrata*, transcription and translation (8%) for *C. parapsilosis*, and stress response for *C. tropicalis* (3%). Among the identified proteins, there were no proteins whose main function will indicate that they are involved in stress response and pathogenesis for *C. parapsilosis*.

**Figure 4:**
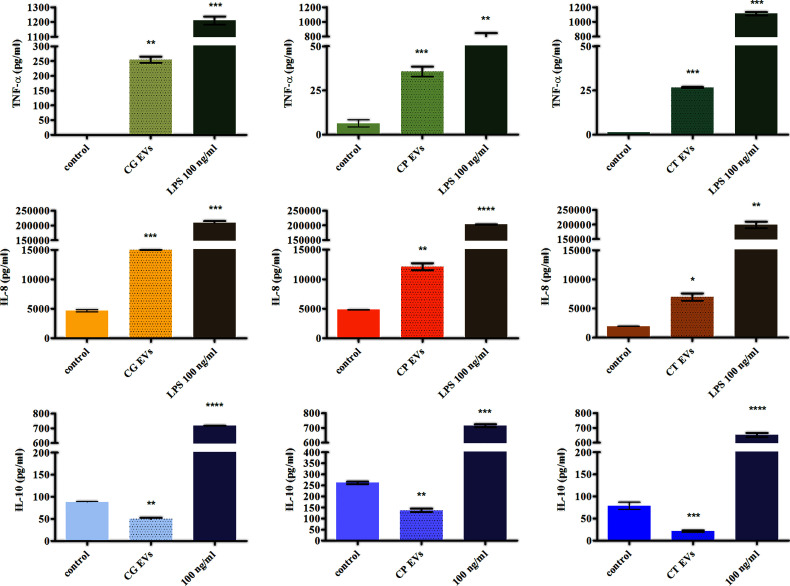
The analysis of cytokine production by THP-1 cells stimulated with *Candida* EVs. The amount of a particular cytokine was shown for stimulation with *C. glabrata* EVs (CG), *C. parapsilosis* EVs (CP), and *C. tropicalis* EVs (CT). A representative result of three independent experiments is presented. To analyze the statistical significance versus control, an unpaired *t*-test was performed with GraphPad Prism software version 7.0 (GraphPad Software, La Jolla, CA, USA). The statistical significance levels were marked with * for *p < *0.05, ** for *p < *0.01, *** for *p < *0.001, and **** for *p* < 0.0001.

Previous reports showed that vesicles produced by *C. albicans* free-floating cells exhibited immunoregulatory properties, inducing membrane expression of co-stimulatory molecules by the host’s immune cells and influencing the production of cytokines (Vargas et al., 2015; Vargas et al., 2020; Martínez-López et al., 2022). There is still a lack of studies on the impact of candidal biofilm-originated vesicles on the host response, and the influence of EVs released by *C. glabrata*, *C. parapsilosis*, and *C. tropicalis* cells existing in the form of sedentary fungal communities has not yet been investigated. In the current study, we examined the effect of these particles on cytokine production by cells from the human monocytic cell line THP-1 differentiated into macrophage-like cells. Analysis of the secretion of proinflammatory and anti-inflammatory cytokines by THP-1 cells stimulated with EVs of each *Candida* species showed a quite comparable tendency (**Figure 4**). There has been a significant increase in the level of TNF-α production after contact of human cells with EVs released by the tested *Candida* fungi. The highest increase in the production of this cytokine, compared with the control, was observed after stimulation with EVs produced by *C. glabrata* biofilm, and less amount of cytokine was produced by the other two species. Similarly, the level of IL-8 secretion after incubation of human cells with candidal EVs was higher than the level for cells not stimulated with EVs, and the highest amount of cytokine was again produced under the influence of *C. glabrata* EVs. On the contrary, the decrease in the amount of cytokine produced was observed for IL-10 after incubation of THP-1 cells with EVs from each tested *Candia* species, when compared with non-stimulated cells.

**Figure 5:**
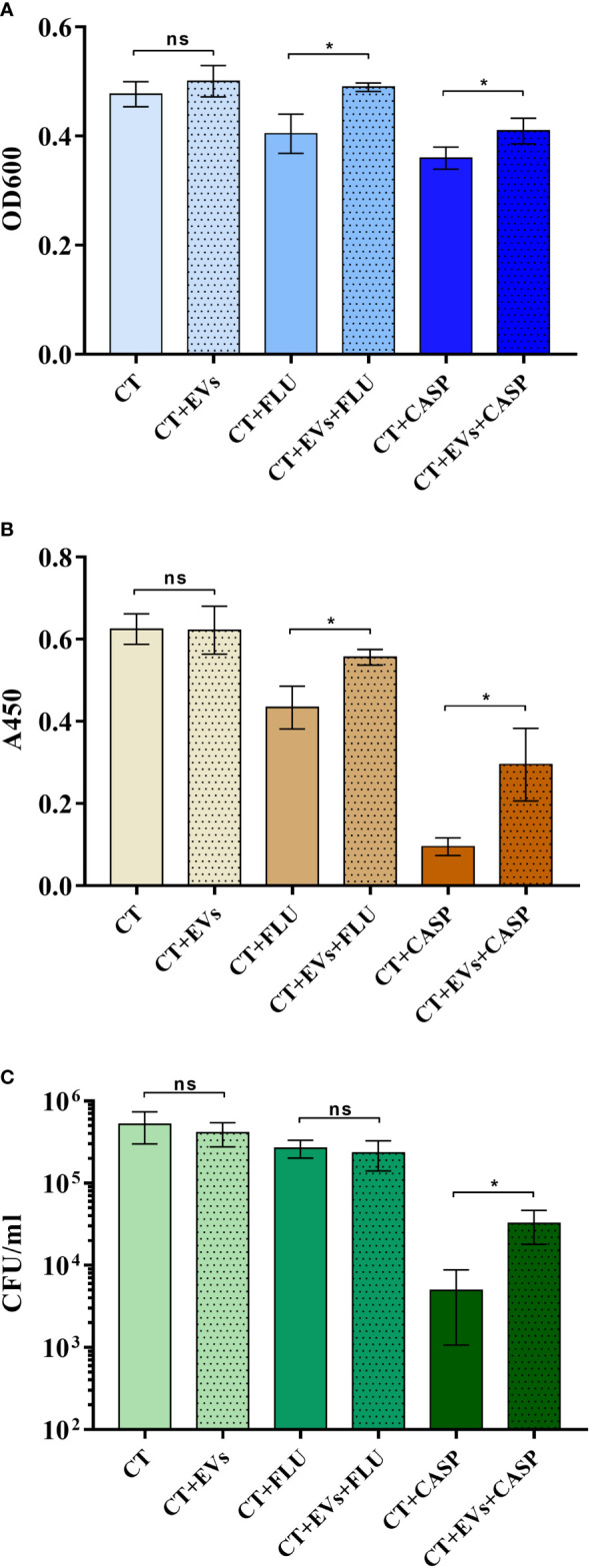
*Candida tropicalis* biofilm formation in the presence of *C. tropicalis* biofilm-derived EVs and the antifungal drugs fluconazole (FLU) and caspofungin (CASP). The thickness of the biofilm was investigated by measuring optical density at 600 nm in the area scan mode **(A)**, the metabolic activity of cells in the biofilm by the XTT test **(B)**, and the viability of the cell by counting CFUs **(C)**. A representative result of three independent experiments is presented. To analyze the statistical significance between samples, an unpaired *t*-test was performed with GraphPad Prism software version 7.0. The statistical significance levels were marked with * for *p < *0.05 and ns when not significant.

In the case of *C. albicans* biofilm, it has been shown that vesicles produced by cells in the biofilm contribute to the formation of the biofilm matrix and the occurrence of fluconazole resistance (Zarnowski et al., 2018; Zarnowski et al., 2021). Therefore, in this study we examined whether EVs might influence formation of biofilm by C. tropicalis, which of the three tested Candida species produced the most stable biofilm and was susceptible to the applied antifungal drugs ‒ fluconazole (FLU) and caspofungin (CASP) (**Figure 5**).

Antifungals were used at concentrations of 0.5 µg/ml for fluconazole and 0.01 µg/ml for caspofungin (Bizerra et al., 2008; Ferreira et al., 2009). In the first step, 10^5^ cells were added to the wells of a microplate and incubated for 90 min to allow them to adhere to the surface. After this time, the non-adherent cells were washed away and the adherent cells were further incubated with or without vesicles produced by the 48-h *C. tropicalis* biofilm. Additionally, after preincubation of the adherent cells with EVs for 90 min, the antifungal drugs fluconazole or caspofungin were added to particular wells, and biofilms were further formed for 24 h, all the time in the presence of EVs where they were originally introduced. Reference samples were biofilms formed without EVs added. To investigate the impact of EVs on the thickness of biofilm formed by *C. tropicalis* cells in the presence of antifungals, optical density measurements in the area scan mode (OD_600_) were performed (**Figure 5A**). Biofilm metabolic activity and cell viability were analyzed with the XTT test (**Figure 5B**) and CFU counting (**Figure 5C**), respectively. It was indicated that in the presence of both antifungal drugs, the addition of EVs increased the thickness of the biofilm and the metabolic activity of the cells, as well as cell viability (only for caspofungin), when compared with samples where EVs were not added; however, in the absence of drugs, the addition of EVs did not significantly affect these parameters under the conditions tested.

## Discussion

Fungi from the *Candida* genus, including *C. glabrata*, *C. parapsilosis*, and *C. tropicalis*, are important commensal pathogens able to cause various types of infections in humans. Although currently these species are not as common cause of fungal diseases as C. albicans, they pose a growing threat to the immunocompromised patient population due to the everincreasing frequency of infections and emerging resistance to antifungals. During the colonization of different niches of the human organism, *Candida* yeasts exploit many various mechanisms of virulence, including the ability to form biofilm and the production of EVs (Yu et al., 1994; Zuza-Alves et al., 2017). EVs are nanometric structures surrounded by a lipid bilayer and filled with a plenteous and assorted cargo (Joffe et al., 2016). These structures have become more widely described in the last few decades primarily for human cells (Faure et al., 2006; Bobis-Wozowicz et al., 2017; You et al., 2020), but also for numerous bacteria (Knox et al., 1967; Lee et al., 2009; Rivera et al., 2010; Tashiro et al., 2010; Lee et al., 2013; Brown et al., 2014; Olaya-Abril et al., 2014), as well as for some fungi (Albuquerque et al., 2008; Vallejo et al., 2012; Wolf et al., 2014; Vargas et al., 2015; Joffe et al., 2016; de Toledo Martins et al., 2018; Zamith-Miranda et al., 2018). Nonetheless, fungi are the least studied so far. There are many possible functions that fungal EVs might perform, which are suggested on the basis of published but still limited research. One of the proposed functions of candidal vesicles is acting as carriers of virulence factors and signaling molecules for intercellular communication (Oliveira et al., 2010). EVs and their content might participate in the interaction between fungal pathogens and human cells during infection development, inducing also immune system response (Gil-Bona et al., 2015; de Toledo Martins et al., 2018). Moreover, *Candida* EVs might also play an important role in the communication between cells of the same species forming a complex community, influencing fungal cell adhesion to different surfaces, formation of biofilm structures, and further development and propagation of infection (Zarnowski et al., 2018; Zarnowski et al., 2021). In addition, the communication through EV release between *Candida* yeasts and other microorganisms living in the same niche might be crucial in the development of multispecies infections.

EVs might be produced by yeast cells grown in different morphological forms. Previously, we described the population of EVs released by planktonic cells and free-floating aggregates of non*-albicans Candida* species (Karkowska-Kuleta et al., 2020), consisting of several subpopulations of vesicles differing in size. In the current study, the heterogeneity of biofilm vesicle population has been demonstrated. *Candida glabrata* EVs produced by biofilm structures were rather smaller in diameter than the ones produced by planktonic cells. For *C. tropicalis*, the sizes were similar when comparing both forms, and in the case of *C. parapsilosis*, the mean sizes of biofilm EVs were larger than those determined by the NTA for the EVs released by free-floating cells (Karkowska-Kuleta et al., 2020). The dimensions of *C. parapsilosis*, *C. glabrata*, and *C. tropicalis* biofilm-derived vesicles were in a similar size range compared with previously described EVs released by *C. albicans* biofilm, constituting a population of structures of 30–200 nm in diameter (Zarnowski et al., 2018), although such smallest particles have not been measured for NAC species. The concentration of proteins and lipids was higher in EVs released by *C. glabrata* biofilm than in those produced by planktonic cells. In the case of *C. parapsilosis* EVs, the opposite was true and higher concentrations of proteins and lipids were proven for vesicles released by planktonic cells, whereas *C. tropicalis* biofilm vesicles contained a higher lipid content, and the protein concentration was comparable for both forms. The concentration of vesicular carbohydrates was not analyzed in a previous study; however, the presence of surface-exposed mannoproteins for EVs produced by planktonic fungal cells was demonstrated with flow cytometry (Karkowska-Kuleta et al., 2020). The current results indicate that in the case of biofilm-originated EVs, the highest concentration of polysaccharides was observed for *C. tropicalis*, while in the other two tested *Candida* species, the carbohydrate concentrations were rather within a similar range.

If the abundance of protein categories in planktonic vesicles (Karkowska-Kuleta et al., 2020) and biofilm vesicles is compared, for *C. parapsilosis*, a depletion of proteins related to pathogenesis is noticeable. However, for all species, a large proportion of the proteins are not well recognized and have no assigned functions in the database; hence, the actual categorization might be slightly different. In the case of *C. glabrata*, the presence of hydrolytic enzymes related to the adhesive properties and virulence of this species, i.e., yapsin 1 aspartyl proteinase (Yps1) and phospholipase 1 (Plb1), is noteworthy (Kaur et al., 2007; Gómez-Molero et al., 2015). Interestingly, in the case of *C. glabrata* and *C. parapsilosis*, two proteins from the SUR7 protein family have been identified, while for *C. albicans*, representatives of this family have been indicated as markers for candidal EVs (Dawson et al., 2020). Despite the fact that the determined concentration of vesicular proteins was the highest for *C. tropicalis*, the greatest protein diversity was observed for EVs of *C. glabrata*, and also with regard to their functionality, where each of the distinguished categories was represented, and the most numerous was the group of proteins related to cellular metabolism and transcription and translation. Moreover, the group of proteins responsible for cell wall organization, along with proteins involved in membrane transport, was represented in each species, which certainly highlights the role of fungal vesicles in the organization of this outer compartment of the cell and, in the case of the currently studied structures, most likely also in the formation of the biofilm matrix. The role of vesicles in building the biofilm matrix was confirmed previously for *C. albicans* (Zarnowski et al., 2018); additionally, the involvement of particular proteins constituting the cargo of biofilm EVs in biofilm regulation has been demonstrated, including the cell wall protein Ecm33 and the cell surface mannoprotein Mp65 (Zarnowski et al., 2021). The ortholog of the Ecm33 protein in our study was identified in both *C. glabrata* and *C. parapsilosis*, while Mp65 was identified for *C. parapsilosis* and *C. tropicalis*. The latter protein in *C. albicans* is a well-known antigen located at the cell wall, mainly of the hyphae form, stimulating T-cell proliferation of human peripheral blood mononuclear cells and inducing dendritic cell maturation and their production of proinflammatory cytokines—TNF-α and IL-6 (Gomez et al., 1996; Nisini et al., 2001; Pietrella et al., 2006).

Vesicles as extracellularly secreted and biologically active structures might participate in the communication between fungi and human cells inducing the immune system response (Herkert et al., 2019; Munhoz da Rocha et al., 2020). For EVs released by *C. albicans* planktonic cells, a few studies were presented previously showing the ambiguous character of the triggered response (Vargas et al., 2015; Vargas et al., 2020; Zamith-Miranda et al., 2021). Thus far, the effect of vesicles on changes in the production of several different cytokines has been shown, including TNF-α (one of the major proinflammatory cytokines regulating immune cells), which controls apoptotic pathways and stimulates the further release of other cytokines, or IL-10 (a potent anti-inflammatory interleukin), which suppresses the secretion of proinflammatory cytokines (Zhang and An, 2007). Vargas et al. (2015) showed that both TNF-α and IL-10 were released by murine primary phagocytes and cells from cell lineages in greater amounts after stimulation with fungal EVs than in the control samples, making it challenging to have a clear definition of the type of immune response induced. These studies showed also the increased level of IL-10 produced by murine dendritic cells compared with control without EVs added (Vargas et al., 2015); however, in later studies, this tendency was observed with a lower level of IL-10 compared with the previous result, albeit it was still higher than in the control sample (Vargas et al., 2020). In contrast, in the work of Zamith-Miranda et al. (2021), the production of TNF-α and IL-10 by murine dendritic cells was not detected after their stimulation with EVs produced by *C. albicans* and *C. auris*. In the current study, the immunomodulating properties of EVs of each investigated non-*albicans Candida* species were demonstrated by the evaluation of the level of cytokines released by human THP-1 cells differentiated into macrophage-like cells. The obtained results revealed an increasing tendency in the production of the proinflammatory cytokine TNF-α and a decreasing release of the anti-inflammatory cytokine IL-10 compared with cells untreated with EVs. This might be a signal that *C. glabrata*, *C. tropicalis*, and *C. parapsilosis* biofilm-derived vesicles induce a rather proinflammatory response of the human immune cells, thereby making *Candida* non-*albicans* biofilm vesicles promising components in anti-*Candida* vaccines. Reducing the concentration of IL-10 at the site of infection leads to increased apoptosis and inflammation, and although it accelerates the removal of the microbial factor causing infection, it also triggers significant damage associated with the expansion of the inflammatory state (Vazquez-Torres et al., 1999; Cyktor and Turner, 2011). The observed increase in the IL-8 secretion by THP-1 cells allows us to conclude that the presence of EVs of each tested non-*albicans Candida* species might also stimulate response involving other immune cells, as IL-8 is a chemoattractant affecting neutrophils and stimulating their chemotaxis, degranulation, and cytotoxicity, and the enhanced production of this interleukin is a signal favoring their migration to the infectious niche. Therefore, all these observations indicate rather the stimulation of a defense response of human cells in response to *Candida* non-*albicans* vesicles at the studied contact time.

As previously demonstrated for *C. albicans*, EVs also contribute to the regulation of fungal biofilm (Zarnowski et al., 2018; Zarnowski et al., 2021). The analysis of the formation of biofilm by *C. tropicalis* cells in the presence of EVs derived from the 48-h biofilm culture, introduced at an early stage of biofilm production, corroborated the substantial role of vesicles in the process of drug resistance that occurs in this complex structure of pathogen existence. Although the introduction of vesicles into cells growing without the presence of antifungal drugs did not cause a statistically significant effect on the biofilm (it also developed without EVs), in the presence of caspofungin and fluconazole, the protective effect of the vesicles was noticeable. In the case of the two drugs tested, both the thickness of the biofilm and the metabolic activity of the cells in the biofilm were partially restored if EVs from the mature biofilm were present at the stage of biofilm initiation; similarly, in the presence of caspofungin, cell viability was higher in biofilms supported by the added EVs. In the case of *C. tropicalis* biofilm, this may indicate the involvement of vesicles, among other mechanisms, in resistance to antifungals. Related conclusions were presented previously for the susceptibility of *C. albicans* biofilm to fluconazole, but these were from studies using mutant strains impaired in vesicle secretion and with incomplete cargo (Zarnowski et al., 2018; Zarnowski et al., 2021). With regard to *C. tropicalis*, the mechanisms of the observed phenomenon still require further detailed studies.

The characteristic and immunobiological properties of candidal vesicles investigated in the present study reveal to some extent the complexity of functions performed by fungal biofilm-derived EVs, thus also indicating the need for further research in this area which could result in a better understanding of the mechanism of development and pathogenesis of candidal infections.

## Data Availability Statement

The datasets presented in this study can be found in online repositories. The names of the repository/repositories and accession number(s) can be found below: http://www.proteomexchange.org/, PXD033327; http://www.proteomexchange.org/, PXD033299; and http://www.proteomexchange.org/,PXD033300.

## Author Contributions

KK, JK-K, EK, OW, and MR-K planned the methodology and experiments. KK, JK-K, EK, and OW executed the experiments. PK and AO provided the resources. KK and JK-K wrote the manuscript draft. MR-K, AK, EZ-S, AO, and EP contributed to the editing and preparation of the final version of the manuscript. All authors contributed to the article and approved the submitted version.

## Funding

This work was financially supported by the National Science Centre of Poland (grant no. 2019/33/B/NZ6/02284 awarded to MR**-**K).

## Conflict of Interest

The authors declare that the research was conducted in the absence of any commercial or financial relationships that could be construed as a potential conflict of interest.

## Publisher’s Note

All claims expressed in this article are solely those of the authors and do not necessarily represent those of their affiliated organizations, or those of the publisher, the editors and the reviewers. Any product that may be evaluated in this article, or claim that may be made by its manufacturer, is not guaranteed or endorsed by the publisher.
