# Supplementary material for: Insight Into the Properties and Immunoregulatory Effect of Extracellular Vesicles Produced by Candida glabrata, Candida parapsilosis, and Candida tropicalis Biofilms
Source: Front Cell Infect Microbiol. 2022 Jun 6;12:879237. doi: 10.3389/fcimb.2022.879237 (PMC9207348; doi:10.3389/fcimb.2022.879237)
Supplement: Supplementary file 1 [file Table_1.pdf]

Supplementary Table 1. Mass spectrometry identification of *C. glabrata*, *C. parapsilosis* and *C. tropicalis* proteins in EVs.

Accession Number	Protein Description	Molecular mass [kDa]	Score	Coverage	# Proteins	# Unique Peptides	# Peptides	# PSMs	# AAs	calc. pI
Candida glabrata										
SCV16649.1	probable Plasma membrane ATPase 1 [*Candida glabrata*]	97.5	1436.39	27.18	3	21	21	34	894	4.96
KTA98889.1	Aspartic proteinase 3 [*Candida glabrata*]	58.8	1151.19	21.34	3	12	12	33	539	6.48
KTB16771.1	Cell wall protein CWP1 [*Candida glabrata*]	20.7	997.57	41.51	3	2	7	19	212	7.40
KAH7583423.1	E1-E2 ATPase [*Candida glabrata*]	120.2	977.87	23.09	5	19	19	25	1087	5.36
KAH7588681.1	Glycosyl hydrolases family 17 signature [*Candida glabrata*]	39.4	966.21	31.02	7	9	9	19	374	5.36
CAO91867.1	ABC-transporter [*Candida glabrata*]	170.0	805.79	17.45	7	22	22	22	1507	7.02
KAH7578809.1	hypothetical protein [*Candida glabrata*]	30.1	781.32	32.73	2	6	6	16	278	5.83
KAH7589292.1	Major facilitator superfamily (MFS) profile [*Candida glabrata*]	60.7	697.79	16.48	2	12	12	20	540	6.54
XP_445865.1	uncharacterized protein CAGL0E04092g [*Candida glabrata*]	72.9	620.93	13.64	1	8	8	13	645	7.80
OXB44727.1	hypothetical protein [*Candida glabrata*]	42.0	567.61	25.99	3	9	9	14	377	6.46
KTB10206.1	Flavoprotein-like protein YCP4 [*Candida glabrata*]	29.8	523.27	35.06	3	8	8	13	271	7.02
KTA96251.1	Glyceraldehyde-3-phosphate dehydrogenase 2 [*Candida glabrata*]	35.9	518.01	36.45	6	12	12	15	332	6.96
XP_449113.1	uncharacterized protein CAGL0L07722g [*Candida glabrata*]	44.6	483.22	36.06	2	13	13	14	416	6.83
KAH7579269.1	X8 domain [*Candida glabrata*]	60.4	465.39	15.04	3	5	7	10	565	5.01
KAH7591716.1	60S ribosomal protein L4 C-terminal domain [*Candida glabrata*]	38.9	424.15	38.12	2	12	12	13	362	10.73
SCV15549.1	Enolase 2 [*Candida glabrata*]	46.3	408.54	31.18	5	12	12	14	433	6.00
XP_446490.1	uncharacterized protein CAGL0G02915g [*Candida glabrata*]	35.1	389.06	34.41	5	7	9	10	311	4.77
XP_444860.1	uncharacterized protein CAGL0A02211g [*Candida glabrata*]	61.5	386.10	18.30	12	4	8	12	552	7.83
XP_449164.1	uncharacterized protein CAGL0L08932g [*Candida glabrata*]	35.0	381.00	35.58	1	8	10	11	312	4.86
KTA95215.1	Lysophospholipase 1 [*Candida glabrata*]	145.6	377.62	7.71	9	9	9	11	1349	4.87
XP_446529.1	uncharacterized protein CAGL0G03795g [*Candida glabrata*]	69.5	351.77	20.47	5	4	11	11	640	5.06
XP_447189.1	60S ribosomal protein L1 [*Candida glabrata*]	24.4	338.07	29.95	1	7	7	9	217	9.72
KAH7587524.1	Glucanosyltransferase [*Candida glabrata*]	51.9	335.99	12.29	5	5	5	8	480	4.69
ADO14231.1	beta-1,3-glucan synthase catalytic subunit [*Candida glabrata*]	213.8	335.28	6.49	53	3	10	10	1863	7.81
KAH7582313.1	putative AMP-binding domain signature [*Candida glabrata*]	77.1	324.83	18.39	3	11	12	12	696	8.47
XP_445466.1	uncharacterized protein CAGL0D01188g [*Candida glabrata*]	49.8	323.98	26.86	5	11	11	12	458	9.09
KTB07093.1	SUR7 family protein FMP45 [*Candida glabrata*]	32.0	315.77	24.29	2	6	6	14	280	8.15
XP_446718.1	ubiquitin-40S ribosomal protein S31 fusion protein [*Candida glabrata*]	17.1	312.83	42.76	11	6	6	9	152	9.76
CAC83344.1	1,3-beta-glucanosyltransferase GAS-1 homologue [*Candida glabrata*]	59.9	311.45	15.21	4	4	6	8	559	4.82
KAH7579088.1	Carbohydrate binding domain (family 19) [*Candida glabrata*]	54.6	310.88	12.19	5	5	5	7	517	4.56
OXB48210.1	hypothetical protein [*Candida glabrata*]	26.8	309.41	30.21	4	7	7	9	235	10.40

XP_445957.1	uncharacterized protein CAGL0E06160g [*Candida glabrata*]	32.6	309.32	36.88	1	9	9	10	282	4.92
KAH7582220.1	Glycosyl hydrolase family 65 central catalytic domain [*Candida glabrata*]	136.5	300.72	10.15	10	13	13	14	1212	5.08
XP_445747.1	uncharacterized protein CAGL0E01353g [*Candida glabrata*]	47.9	294.34	13.39	1	4	4	6	433	6.07
SCV14429.1	60S ribosomal protein L7 [*Candida glabrata*]	27.4	287.79	32.64	3	9	9	9	242	10.21
KAH7579246.1	NAD binding domain of 6-phosphogluconate dehydrogenase [*Candida glabrata*]	53.5	281.98	21.27	3	10	10	10	489	6.61
KAH7589698.1	SUR7/PalI family [*Candida glabrata*]	79.7	265.56	10.12	6	6	6	6	741	5.21
KAH7589795.1	hypothetical protein [*Candida glabrata*]	72.7	261.19	10.07	5	4	4	5	675	6.47
KAH7586909.1	Membrane-associating domain [*Candida glabrata*]	28.5	258.51	17.58	3	3	3	4	256	7.23
KAH7589068.1	Protein of unknown function (DUF3533) [*Candida glabrata*]	61.7	255.10	11.93	11	5	6	6	545	7.52
XP_445106.1	uncharacterized protein CAGL0B03069g [*Candida glabrata*]	36.7	253.23	24.55	4	7	7	7	334	6.11
KAH7588511.1	Ribosomal protein L18e signature [*Candida glabrata*]	20.7	243.00	34.41	2	7	7	8	186	11.75
KTB23789.1	Pyruvate kinase 1 [*Candida glabrata*]	54.7	241.99	16.97	5	7	7	7	501	6.48
XP_448731.1	uncharacterized protein CAGL0K11858g [*Candida glabrata*]	21.0	237.22	28.28	1	6	6	8	198	6.54
XP_447969.1	uncharacterized protein CAGL0J06050g [*Candida glabrata*]	37.8	225.83	11.41	1	4	4	6	368	8.66
KTB20581.1	Iron transport multicopper oxidase FET3 [*Candida glabrata*]	72.0	223.82	11.34	3	6	6	7	635	5.05
KAH7580546.1	Cellulase (glycosyl hydrolase family 5) [*Candida glabrata*]	51.1	223.34	15.12	5	7	7	7	443	4.93
AAN77243.1	pyruvate decarboxylase [*Candida glabrata*]	61.7	218.80	12.43	2	6	6	7	563	5.87
OXB44829.1	hypothetical protein CAGL0C00671g [*Candida glabrata*]	89.3	217.49	7.91	9	5	5	5	834	9.35
AAF81925.1	elongation factor 2, partial [*Candida glabrata*]	89.9	214.56	10.44	3	7	7	7	814	6.52
XP_445283.1	uncharacterized protein CAGL0C02453g [*Candida glabrata*]	23.9	212.78	29.82	3	6	6	6	218	5.86
KAH7587075.1	Glycosyl hydrolases family 16 (GH16) domain profile [*Candida glabrata*]	48.0	205.49	15.27	7	6	6	6	452	4.96
XP_445544.1	uncharacterized protein CAGL0D02948g [*Candida glabrata*]	73.3	203.19	9.90	3	5	6	7	667	4.89
XP_447807.1	60S ribosomal protein L2 [*Candida glabrata*]	27.3	202.83	26.77	3	7	7	7	254	10.93
XP_448490.1	40S ribosomal protein S17 [*Candida glabrata*]	15.8	201.80	33.09	3	4	4	4	136	10.59
XP_447419.1	uncharacterized protein CAGL0I03916g [*Candida glabrata*]	20.6	199.33	35.36	3	6	6	6	181	6.58
XP_448200.1	40S ribosomal protein S3 [*Candida glabrata*]	27.1	193.79	31.17	1	8	8	8	247	9.33
XP_449514.1	60S ribosomal protein L25 [*Candida glabrata*]	15.7	193.21	24.65	2	3	3	4	142	10.14
XP_447590.1	uncharacterized protein CAGL0I07843g [*Candida glabrata*]	37.5	187.49	22.44	1	6	6	6	352	6.46
XP_449048.1	uncharacterized protein CAGL0L06270g [*Candida glabrata*]	28.3	186.59	21.69	2	4	5	5	249	4.96
XP_444845.2	uncharacterized protein CAGL0A01826g [*Candida glabrata*]	65.6	174.21	12.24	8	5	6	6	588	7.77
XP_446452.1	60S ribosomal protein L3 [*Candida glabrata*]	43.8	172.29	11.37	1	5	5	8	387	10.27
XP_449381.1	40S ribosomal protein S1 [*Candida glabrata*]	28.7	168.21	17.65	1	5	5	5	255	9.98
OXB41622.1	hypothetical protein CAGL0K07414g [*Candida glabrata*]	20.4	167.59	26.74	2	5	5	5	172	10.35
XP_445788.1	uncharacterized protein CAGL0E02255g [*Candida glabrata*]	13.8	166.30	20.16	1	3	3	4	124	5.14
KTB03478.1	Inorganic phosphate transporter PHO84 [*Candida glabrata*]	63.7	165.73	9.72	5	5	5	5	576	7.09

KTA96608.1	Hyphally regulated cell wall protein 3, partial [*Candida glabrata*]	56.4	165.50	8.99	24	4	4	4	523	4.79
KTB12456.1	Non-classical export protein 2 [*Candida glabrata*]	18.9	163.85	23.26	2	3	3	5	172	9.85
KTB04356.1	Cell wall protein ECM33 [*Candida glabrata*]	43.1	159.85	8.08	2	4	4	5	421	5.35
KAH7582282.1	Ribosomal protein L27e signature [*Candida glabrata*]	15.6	158.13	38.97	2	5	5	5	136	10.27
KAH7586526.1	Iron permease FTR1 family [*Candida glabrata*]	46.1	152.98	9.31	2	4	4	4	408	8.63
XP_448152.1	60S ribosomal protein L16 [*Candida glabrata*]	22.5	146.84	21.00	1	4	4	4	200	10.51
KTB01633.1	60S ribosomal protein L15-A [*Candida glabrata*]	24.5	145.79	17.16	2	3	3	4	204	11.37
AAC31800.2	ATP-binding cassette transporter [*Candida glabrata*]	174.9	141.06	3.96	6	4	5	5	1542	7.49
UCS20289.1	uncharacterized protein CAGL0F08063g [*Candida glabrata*]	60.5	140.06	7.32	7	3	3	3	533	7.53
XP_447888.1	uncharacterized protein CAGL0J04202g [*Candida glabrata*]	11.2	136.87	24.27	1	2	2	2	103	5.02
KTB24192.1	Aquaporin-1, partial [*Candida glabrata*]	28.8	135.39	13.06	6	3	3	3	268	7.06
XP_448879.1	uncharacterized protein CAGL0L02497g [*Candida glabrata*]	39.3	134.67	13.57	1	4	4	4	361	5.77
XP_446360.1	40S ribosomal protein S4 [*Candida glabrata*]	29.4	133.51	16.09	2	5	5	5	261	10.01
XP_449936.1	uncharacterized protein CAGL0M13629g [*Candida glabrata*]	20.7	132.28	22.28	2	4	4	4	184	8.79
XP_446734.1	uncharacterized protein CAGL0G08558g [*Candida glabrata*]	25.2	129.11	17.18	3	3	3	3	227	5.44
XP_448726.1	40S ribosomal protein S11 [*Candida glabrata*]	17.9	128.95	16.67	1	4	4	4	156	10.68
KAH7588975.1	Ribosomal protein S13/S18 [*Candida glabrata*]	14.9	125.32	18.11	3	2	2	2	127	10.42
SCV15493.1	related to Casein kinase I homolog 2 [*Candida glabrata*]	49.5	124.85	6.26	5	2	2	2	447	6.96
XP_448774.1	60S ribosomal protein L10 [*Candida glabrata*]	25.5	123.44	19.91	2	5	5	5	221	10.02
KTA95671.1	High-affinity glutamine permease [*Candida glabrata*]	73.0	123.30	6.21	3	4	4	4	660	6.90
KTB03762.1	Fatty acid synthase subunit alpha [*Candida glabrata*]	199.3	121.28	2.89	6	4	4	4	1802	5.86
KAH7579016.1	putative TOS1-like glycosyl hydrolase (DUF2401) [*Candida glabrata*]	43.6	121.18	9.75	4	3	3	3	400	5.17
XP_447279.1	uncharacterized protein CAGL0I00594g [*Candida glabrata*]	24.3	118.06	18.69	1	4	4	4	214	6.68
XP_446098.1	60S ribosomal protein L12 [*Candida glabrata*]	17.7	116.69	14.55	1	2	2	2	165	9.36
KAH7581057.1	Beta-glucosidase (SUN family) [*Candida glabrata*]	37.8	115.44	16.67	7	5	5	5	366	4.88
XP_447900.1	uncharacterized protein CAGL0J04466g [*Candida glabrata*]	29.3	114.75	11.11	1	2	2	2	261	8.72
KAH7590103.1	Glutathione peroxidases signature 2 [*Candida glabrata*]	18.7	114.34	20.73	2	3	3	3	164	8.12
XP_444862.1	uncharacterized protein CAGL0A02255g [*Candida glabrata*]	30.0	114.17	11.40	1	4	4	4	272	4.98
XP_444824.1	uncharacterized protein CAGL0A01221g [*Candida glabrata*]	31.7	113.60	9.90	3	2	2	2	293	7.21
XP_448259.1	uncharacterized protein CAGL0K00803g [*Candida glabrata*]	11.2	112.82	23.30	1	2	2	2	103	4.93
XP_446276.1	40S ribosomal protein S2 [*Candida glabrata*]	27.3	112.60	18.18	1	5	5	5	253	10.30
XP_444833.1	60S ribosomal protein L24 [*Candida glabrata*]	17.5	112.20	17.42	1	3	3	3	155	11.15
OXB42294.1	hypothetical protein CAGL0J09922g [*Candida glabrata*]	44.1	111.88	9.60	10	4	4	4	427	5.11

QNG15711.1	uncharacterized protein CAGL0K07337g [*Candida glabrata*]	35.1	110.16	5.75	3	2	2	3	313	6.04
SCV17393.1	probable Dicarboxylic amino acid permease [*Candida glabrata*]	67.8	109.23	4.47	4	2	2	2	604	8.38
XP_449129.1	40S ribosomal protein S14 [*Candida glabrata*]	14.3	108.06	25.19	5	3	3	3	135	10.86
OXB43926.1	hypothetical protein CAGL0F08481g [*Candida glabrata*]	80.0	105.41	6.99	3	4	4	4	701	7.02
SCV17535.1	related to Cell wall mannoprotein HSP150 [*Candida glabrata*]	15.1	104.11	19.44	10	2	3	3	144	7.80
KAH7581024.1	Malate synthase [*Candida glabrata*]	63.1	103.89	8.48	3	5	5	5	554	6.81
KTA99655.1	Cell wall mannoprotein PIR1, partial [*Candida glabrata*]	20.2	103.08	13.27	8	2	3	3	196	5.48
SCV13687.1	related to Monoglyceride lipase [*Candida glabrata*]	32.1	102.80	9.19	3	2	2	2	283	9.10
XP_445966.1	uncharacterized protein CAGL0E06358g [*Candida glabrata*]	27.6	101.71	10.93	1	3	3	3	247	7.06
XP_446019.1	40S ribosomal protein S15 [*Candida glabrata*]	16.1	100.52	16.78	1	2	2	3	143	10.46
OXB44630.1	hypothetical protein CAGL0D02442g [*Candida glabrata*]	48.1	99.42	7.91	3	3	3	3	417	6.90
XP_444864.1	uncharacterized protein CAGL0A02299g [*Candida glabrata*]	31.1	98.87	10.04	7	3	3	3	279	7.84
XP_449631.1	60S ribosomal protein L21 [*Candida glabrata*]	18.1	97.55	10.63	3	2	2	2	160	10.40
KTB00636.1	Ornithine aminotransferase [*Candida glabrata*]	46.9	94.00	6.50	3	2	2	2	431	6.48
XP_446638.1	uncharacterized protein CAGL0G06358g [*Candida glabrata*]	12.7	92.15	20.35	1	2	2	2	113	7.14
KAH7579012.1	Ribosomal protein L22p/L17e [*Candida glabrata*]	11.9	91.94	9.35	4	1	1	2	107	10.43
KAH7588962.1	hypothetical protein [*Candida glabrata*]	78.1	89.07	5.17	4	3	3	3	696	6.64
SCV13642.1	probable ATP-dependent molecular chaperone HSC82 [*Candida glabrata*]	79.6	88.68	4.76	4	3	3	3	694	4.84
KAH7591060.1	hypothetical protein [*Candida glabrata*]	100.4	88.45	2.56	3	2	2	2	900	8.62
XP_447845.1	40S ribosomal protein S24 [*Candida glabrata*]	15.3	87.17	17.78	2	2	2	2	135	10.58
KAH7585019.1	Late exocytosis, associated with Golgi transport [*Candida glabrata*]	111.1	85.49	3.49	7	3	3	3	974	7.31
OXB45250.1	hypothetical protein [*Candida glabrata*]	37.0	84.69	11.21	3	3	3	3	339	5.34
XP_449076.2	60S ribosomal protein L13 [*Candida glabrata*]	22.6	84.42	10.55	2	2	2	2	199	10.81
KAH7590064.1	Ferredoxin reductase-type FAD binding domain profile [*Candida glabrata*]	33.6	83.70	6.38	2	2	2	2	298	8.94
XP_447161.1	uncharacterized protein CAGL0H08327g [*Candida glabrata*]	26.9	83.33	6.45	1	1	1	1	248	6.25
XP_447288.1	40S ribosomal protein S16 [*Candida glabrata*]	15.8	81.77	24.48	1	3	3	3	143	10.32
XP_449144.1	uncharacterized protein CAGL0L08448g [*Candida glabrata*]	19.4	81.08	8.09	1	2	2	3	173	9.17
XP_448317.1	40S ribosomal protein S26 [*Candida glabrata*]	13.5	80.98	20.17	1	2	2	2	119	11.02
KTB02189.1	hypothetical protein [*Candida glabrata*]	11.4	80.38	11.21	4	1	1	1	107	7.15

XP_448482.1	uncharacterized protein CAGL0K05973g [*Candida glabrata*]	60.4	79.73	2.11	1	1	1	1	568	5.22
SCV14710.1	Elongation factor 3 [*Candida glabrata*]	114.8	79.41	1.93	4	3	3	3	1038	6.00
KAH7580740.1	Cobalamin-independent synthase, N-terminal domain [*Candida glabrata*]	85.9	78.88	1.96	5	2	2	2	767	6.46
SCV15271.1	40S ribosomal protein S25 [*Candida glabrata*]	10.4	78.32	21.98	2	2	2	2	91	10.29
SCV17114.1	probable Flavin carrier protein 2 [*Candida glabrata*]	85.3	78.24	2.37	5	2	2	2	760	8.57
KAH7589703.1	N-terminal half of MaoC dehydratase [*Candida glabrata*]	233.5	78.05	1.15	6	2	2	2	2081	5.64
KAH7589738.1	Transketolase signature 2 [*Candida glabrata*]	73.6	77.75	5.60	2	3	3	3	679	6.46
KTA95896.1	Leucine--tRNA ligase, cytoplasmic [*Candida glabrata*]	124.5	75.80	2.38	3	2	2	2	1093	5.77
AAM74217.1	HHT2p [*Candida glabrata*]	15.3	75.50	10.29	2	2	2	3	136	11.43
OXB43656.1	hypothetical protein [*Candida glabrata*]	23.7	73.30	10.70	2	2	2	2	215	8.21
XP_002999536.1	uncharacterized protein CAGL0E02315g [*Candida glabrata*]	14.2	72.14	12.03	1	1	2	2	133	10.62
XP_448104.1	60S ribosomal protein L35 [*Candida glabrata*]	14.1	72.11	17.50	4	2	2	2	120	10.65
QNG13690.1	uncharacterized protein CAGL0F08833g [*Candida glabrata*]	96.8	71.19	1.08	6	1	1	1	922	4.40
XP_446291.1	uncharacterized protein CAGL0F07447g [*Candida glabrata*]	13.7	70.44	10.57	1	1	1	1	123	9.57
KAH7591726.1	hypothetical protein [*Candida glabrata*]	31.9	70.20	4.24	4	1	1	1	283	7.64
XP_444910.1	60S ribosomal protein L19 [*Candida glabrata*]	21.7	69.68	14.29	4	3	3	3	189	11.50
KAH7589444.1	Glucanosyltransferase [*Candida glabrata*]	57.1	69.06	4.21	4	2	2	2	523	5.40
KAH7584236.1	Eukaryotic mitochondrial porin signature [*Candida glabrata*]	30.3	68.37	8.13	3	2	2	2	283	8.69
KTA95589.1	Citrate synthase, mitochondrial [*Candida glabrata*]	50.6	67.71	2.43	4	1	1	1	452	8.50
SCV12659.1	S-adenosylmethionine synthase 2 [*Candida glabrata*]	41.2	67.52	4.02	5	1	1	1	373	5.21
XP_446440.1	60S ribosomal protein L11 [*Candida glabrata*]	19.8	67.49	8.62	1	2	2	2	174	10.15
XP_444964.1	40S ribosomal protein S8 [*Candida glabrata*]	22.4	66.92	8.46	3	2	2	2	201	10.65
XP_446991.1	60S ribosomal protein L32 [*Candida glabrata*]	14.8	65.98	13.74	1	2	2	2	131	10.99
XP_448356.1	uncharacterized protein CAGL0K03025g [*Candida glabrata*]	18.7	65.30	8.38	1	1	1	2	167	7.30
XP_446000.1	uncharacterized protein CAGL0F00605g [*Candida glabrata*]	54.9	63.98	3.84	1	2	2	2	495	5.57
XP_444894.1	uncharacterized protein CAGL0A02948g [*Candida glabrata*]	22.1	62.70	5.79	1	1	1	1	190	5.05
KTB27062.1	putative family 17 glucosidase SCW11 [*Candida glabrata*]	39.0	62.52	3.49	13	1	1	1	373	5.16
XP_448749.1	uncharacterized protein CAGL0K12276g [*Candida glabrata*]	21.6	62.36	8.25	1	2	2	2	194	8.94
XP_449292.1	uncharacterized protein CAGL0L12056g [*Candida glabrata*]	28.3	62.25	7.20	1	1	2	2	250	4.82

XP_445366.1	uncharacterized protein CAGL0C04389g [*Candida glabrata*]	14.0	62.20	6.98	3	1	1	2	129	10.07
XP_445905.1	60S ribosomal protein L9 [*Candida glabrata*]	21.6	61.30	6.28	2	1	1	1	191	9.61
KTB09360.1	Mannose-1-phosphate guanyltransferase 1 [*Candida glabrata*]	39.3	60.98	9.14	3	2	2	2	361	6.13
KTB15431.1	Reduced viability upon starvation protein 167 [*Candida glabrata*]	48.7	60.58	2.72	5	1	1	1	441	5.21
XP_447084.1	uncharacterized protein CAGL0H06633g [*Candida glabrata*]	60.3	60.38	2.57	1	1	1	1	544	6.16
XP_445367.1	uncharacterized protein CAGL0C04411g [*Candida glabrata*]	13.9	59.44	12.21	3	1	2	2	131	10.64
KTA95355.1	Phosphatidylinositol 4,5-bisphosphate-binding protein SLM1 [*Candida glabrata*]	71.8	59.42	2.88	11	2	2	2	625	7.69
KAH7591953.1	Hexokinase [*Candida glabrata*]	53.7	59.30	1.65	4	1	1	1	486	5.54
KAH7586819.1	SH3 domain [*Candida glabrata*]	100.6	59.10	2.07	14	2	2	2	918	9.13
KAH7586725.1	PH domain profile [*Candida glabrata*]	59.7	59.09	1.50	5	1	1	1	532	9.06
KAH7582467.1	Ribosomal protein L36e signature [*Candida glabrata*]	10.9	58.98	12.24	2	1	1	1	98	11.59
KAH7588551.1	hypothetical protein [*Candida glabrata*]	52.7	58.70	3.03	4	2	2	2	495	4.91
SCV17570.1	related to Multiprotein-bridging factor 1 [*Candida glabrata*]	6.3	58.20	43.10	2	2	2	2	58	9.99
XP_449882.1	60S ribosomal protein L34 [*Candida glabrata*]	14.5	57.52	11.72	1	2	2	2	128	10.86
KAH7609587.1	Lecithin:cholesterol acyltransferase [*Candida glabrata*]	79.6	57.05	1.41	4	1	1	1	708	7.40
KTB23845.1	ATP synthase subunit alpha, mitochondrial, partial [*Candida glabrata*]	42.0	56.95	3.57	2	2	2	2	392	8.98
QNG13742.1	RPP0 [*Candida glabrata*]	33.6	56.79	7.07	2	2	2	2	311	4.91
KAH7590574.1	Amino acid permeases signature [*Candida glabrata*]	64.6	56.46	1.92	5	1	1	1	572	7.65
KAH7591435.1	hypothetical protein [*Candida glabrata*]	29.7	56.22	4.85	2	1	1	1	268	5.30
OXB41625.1	hypothetical protein [*Candida glabrata*]	62.9	55.34	3.17	2	2	2	2	567	5.72
XP_002999595.1	40S ribosomal protein S22 [*Candida glabrata*]	14.6	55.11	12.31	1	1	1	1	130	9.99
KAH7585102.1	hypothetical protein [*Candida glabrata*]	57.0	55.10	2.53	7	1	1	1	513	8.51
XP_448732.1	uncharacterized protein CAGL0K11880g [*Candida glabrata*]	35.1	54.95	6.33	3	2	2	2	316	9.29
KAH7581208.1	Ribosomal protein L6e signature [*Candida glabrata*]	20.0	54.84	6.82	3	1	1	1	176	10.17
KAH7586313.1	Glycolipid 2-alpha-mannosyltransferase [*Candida glabrata*]	54.1	54.21	4.10	3	2	2	2	463	5.67
SCV13934.1	40S ribosomal protein S5 [*Candida glabrata*]	24.2	54.04	8.37	3	2	2	2	215	9.32
XP_449464.1	60S ribosomal protein L5 [*Candida glabrata*]	33.7	53.97	6.40	1	2	2	2	297	6.90
KAH7587672.1	Beta-glucan synthesis-associated protein (SKN1) [*Candida glabrata*]	77.5	53.73	1.73	4	1	1	1	693	4.94
XP_445933.1	uncharacterized protein CAGL0E05632g [*Candida glabrata*]	66.2	53.65	3.85	1	2	2	2	598	8.06

KTB10183.1	Nitrosoguanidine resistance protein SNG1 [*Candida glabrata*]	132.7	53.50	1.72	8	1	2	2	1162	8.57
UCS19894.1	uncharacterized protein CAGL0E05258g [*Candida glabrata*]	12.2	52.93	10.19	5	1	1	1	108	7.18
QNG13121.1	YPS11 [*Candida glabrata*]	55.5	52.83	2.17	2	1	1	1	508	5.40
XP_447445.1	uncharacterized protein CAGL0I04510g [*Candida glabrata*]	12.9	51.65	14.66	1	1	1	2	116	5.86
XP_446201.1	uncharacterized protein CAGL0F05269g [*Candida glabrata*]	21.4	51.28	5.76	1	1	1	2	191	6.76
KAH7599938.1	putative AMP-binding domain signature [*Candida glabrata*]	77.6	51.15	2.30	4	1	2	2	696	7.33
KAH7581742.1	Major Facilitator Superfamily [*Candida glabrata*]	72.6	50.89	1.84	2	1	1	1	651	6.60
KAH7579068.1	Serine/Threonine protein kinases active-site signature [*Candida glabrata*]	130.6	49.49	1.22	15	2	2	2	1144	7.15
KTA95369.1	Valine--tRNA ligase, mitochondrial [*Candida glabrata*]	120.4	48.90	2.17	4	2	2	2	1061	6.30
XP_445505.1	uncharacterized protein CAGL0D02090g [*Candida glabrata*]	30.1	48.80	3.61	3	1	1	1	277	6.30
AAM74218.1	IPP1p, partial [*Candida glabrata*]	14.5	48.52	6.82	2	1	1	1	132	5.01
KAH7580749.1	Protein of unknown function (DUF3602) [*Candida glabrata*]	22.3	48.47	4.88	3	1	1	1	205	10.23
KAH7591138.1	tRNA synthetases class I (E and Q), anti-codon binding domain [*Candida glabrata*]	80.3	48.36	2.98	3	2	2	2	705	6.83
XP_447518.1	uncharacterized protein CAGL0I06160g [*Candida glabrata*]	23.8	48.21	3.43	1	1	1	1	233	7.96
KAH7578850.1	Slx4 endonuclease [*Candida glabrata*]	82.2	48.04	1.38	78	2	2	2	726	5.10
SCV16006.1	related to Glycerol uptake/efflux facilitator protein [*Candida glabrata*]	70.4	47.13	6.77	5	2	2	2	635	6.80
KAH7579040.1	FAS1/BIgH3 domain profile [*Candida glabrata*]	28.4	46.94	2.77	3	1	1	1	253	5.67
SCV17244.1	uncharacterized protein [*Candida glabrata*]	45.3	46.94	5.38	4	1	1	1	409	8.82
AVR51203.1	yapsin 1 [*Candida glabrata*]	63.8	46.84	1.33	7	1	1	1	601	5.43
XP_447838.1	uncharacterized protein CAGL0J03080g [*Candida glabrata*]	18.9	46.80	4.46	1	1	1	1	157	6.37
KAH7579029.1	hypothetical protein [*Candida glabrata*]	21.9	46.58	7.14	4	1	1	1	210	5.03
KAH7585159.1	DnaJ domain [*Candida glabrata*]	43.5	45.68	3.66	2	1	1	1	382	5.67
OXB45323.1	hypothetical protein [*Candida glabrata*]	58.7	45.60	1.88	4	1	1	1	531	8.59
XP_447438.1	uncharacterized protein CAGL0I04356g [*Candida glabrata*]	44.8	45.53	2.78	1	1	1	1	396	5.10
XP_448361.1	40S ribosomal protein S20 [*Candida glabrata*]	13.5	43.86	9.24	1	1	1	1	119	9.74
KAH7588113.1	ADP-ribosylation factor family [*Candida glabrata*]	20.6	43.36	6.01	2	1	1	1	183	5.58
KAH7579173.1	Thioredoxin domain profile [*Candida glabrata*]	18.9	42.72	6.29	2	1	1	1	175	5.53
XP_448803.1	uncharacterized protein CAGL0L00671g [*Candida glabrata*]	59.6	42.52	1.84	1	1	1	1	543	5.64
XP_446408.1	60S ribosomal protein L26 [*Candida glabrata*]	14.2	42.33	12.60	2	2	2	2	127	10.55

XP_445776.1	40S ribosomal protein S19 [*Candida glabrata*]	15.8	42.21	7.64	1	1	1	1	144	9.44
XP_446644.1	40S ribosomal protein S7 [*Candida glabrata*]	21.5	42.18	3.19	1	1	1	1	188	9.88
KAH7585244.1	Phosphopantetheine attachment site [*Candida glabrata*]	13.2	42.00	5.13	4	1	1	2	117	5.31
KAH7583679.1	PH domain [*Candida glabrata*]	110.0	40.76	1.79	8	1	1	1	1003	9.32
OXB42905.1	hypothetical protein [*Candida glabrata*]	22.1	40.57	7.73	5	2	2	2	194	9.98
KAH7590988.1	Reticulon domain profile [*Candida glabrata*]	33.0	40.38	3.06	4	1	1	1	294	8.76
XP_445035.1	uncharacterized protein CAGL0B01507g [*Candida glabrata*]	46.8	40.24	1.41	8	1	1	1	427	8.57
KTB00169.1	Guanine nucleotide-binding protein alpha-2 subunit [*Candida glabrata*]	50.6	39.27	2.46	2	1	1	1	448	5.73
KTB11453.1	Zuotin [*Candida glabrata*]	49.1	39.22	2.77	2	1	1	1	433	8.62
KAH7583997.1	RWD domain [*Candida glabrata*]	161.9	39.20	0.35	9	1	1	1	1420	8.73
KTB11426.1	Cell wall integrity sensor MID2 [*Candida glabrata*]	23.6	38.73	4.21	8	1	1	1	214	6.10
KAH7580864.1	Amino acid permeases signature [*Candida glabrata*]	70.4	38.68	1.26	3	1	1	1	634	7.71
XP_446153.1	uncharacterized protein CAGL0F04191g [*Candida glabrata*]	10.6	38.44	10.00	1	1	1	1	90	8.85
QNG15951.1	uncharacterized protein CAGL0K12958g [*Candida glabrata*]	39.7	38.06	1.38	2	1	1	1	362	6.71
KTB24655.1	Splicing factor MUD2 [*Candida glabrata*]	57.5	37.87	1.19	12	1	1	1	505	6.61
KAH7586079.1	Late exocytosis, associated with Golgi transport [*Candida glabrata*]	104.0	37.86	0.87	6	1	1	1	918	7.65
KTB00458.1	putative membrane protein, partial [*Candida glabrata*]	13.4	37.78	10.34	8	1	1	1	116	9.77
KAH7581005.1	Plasma-membrane choline transporter [*Candida glabrata*]	62.6	37.67	1.08	4	1	1	1	557	7.53
XP_447616.1	uncharacterized protein CAGL0I08459g [*Candida glabrata*]	23.1	37.09	3.81	1	1	1	1	210	6.99
XP_446623.1	uncharacterized protein CAGL0G06006g [*Candida glabrata*]	12.4	36.58	10.00	1	1	1	1	110	5.73
KAH7580841.1	Mu homology domain (MHD) profile [*Candida glabrata*]	90.4	36.29	1.11	6	1	1	1	811	8.59
XP_449198.1	uncharacterized protein CAGL0L09801g [*Candida glabrata*]	37.5	36.22	1.57	8	1	1	1	319	6.01
KTA96363.1	Mitochondrial outer membrane protein OM45 [*Candida glabrata*]	48.7	36.01	2.84	3	1	1	1	423	5.64
KAH7583704.1	Cyclophilin-type peptidyl-prolyl cis-trans isomerase signature [*Candida glabrata*]	41.6	35.81	1.62	4	1	1	1	371	5.86
KTB09800.1	Acetyl-coenzyme A synthetase 1 [*Candida glabrata*]	79.2	35.76	1.28	2	1	1	1	704	6.33
XP_446620.2	60S ribosomal protein L42 [*Candida glabrata*]	12.1	35.37	7.55	3	1	1	1	106	10.51
XP_448309.1	uncharacterized protein CAGL0K01925g [*Candida glabrata*]	50.6	35.31	3.13	1	1	1	1	448	5.99
KTB26313.1	Cell wall protein IFF4, partial [*Candida glabrata*]	66.8	34.79	1.79	17	1	1	1	613	5.24
KAH7588589.1	Major intrinsic protein [*Candida glabrata*]	68.1	34.75	2.82	6	1	1	1	602	6.87

KAH7590915.1	Phosphoribosyl-ATP pyrophosphohydrolase [*Candida glabrata*]	88.1	34.18	0.87	7	1	1	1	802	5.25
KAH7584137.1	ANTH domain [*Candida glabrata*]	107.3	34.17	0.74	4	1	1	1	952	5.45
XP_448306.1	rRNA methyltransferase NOP1 [*Candida glabrata*]	34.1	33.81	2.48	1	1	1	1	323	10.13
XP_449792.1	60S ribosomal protein L14 [*Candida glabrata*]	15.2	33.45	8.03	1	1	1	1	137	11.09
XP_448712.1	uncharacterized protein CAGL0K11418g [*Candida glabrata*]	24.4	33.35	4.95	1	1	1	1	222	6.15
AAK15002.1	unknown, partial [*Candida glabrata*]	67.9	33.14	1.03	5	1	1	1	585	6.65
KAH7582402.1	Zinc finger C2H2 type domain signature [*Candida glabrata*]	42.4	32.79	1.52	1	1	1	1	396	9.26
OXB42184.1	hypothetical protein [*Candida glabrata*]	39.9	32.74	2.62	2	1	1	1	344	8.54
KAH7582004.1	Hydantoinase/oxoprolinase [*Candida glabrata*]	141.5	32.46	0.39	6	1	1	1	1280	6.70
XP_447316.1	uncharacterized protein CAGL0I01408g [*Candida glabrata*]	11.5	32.41	5.77	1	1	1	1	104	9.58
KAH7589341.1	hypothetical protein [*Candida glabrata*]	20.3	32.18	3.83	6	1	1	1	209	6.52
SCV15654.1	related to Flavin carrier protein 1 [*Candida glabrata*]	89.9	31.96	1.91	4	1	1	1	786	7.42
KAH7586918.1	Glycogen recognition site of AMP-activated protein kinase [*Candida glabrata*]	85.5	31.88	2.20	10	1	1	1	771	6.05
KAH7590147.1	zinc-RING finger domain [*Candida glabrata*]	40.0	31.58	1.71	5	1	1	1	350	9.45
XP_444832.1	60S ribosomal protein L30 [*Candida glabrata*]	11.4	31.28	10.48	3	1	1	1	105	9.85
ABE27290.1	mitochondrial F-ATPase beta subunit, partial [*Candida glabrata*]	36.1	31.07	3.26	5	1	1	1	337	5.07
XP_447317.1	uncharacterized protein CAGL0I01430g [*Candida glabrata*]	17.2	30.84	10.83	1	1	1	1	157	5.05
XP_448038.1	uncharacterized protein CAGL0J07612g [*Candida glabrata*]	57.0	30.79	1.80	1	1	1	1	500	6.21
KAH7591442.1	AICARFT/IMPCHase bienzyme [*Candida glabrata*]	64.9	30.78	1.02	2	1	1	1	590	6.34
XP_445271.1	uncharacterized protein CAGL0C02189g [*Candida glabrata*]	49.2	30.77	2.45	1	1	1	1	449	5.71
QNG16387.1	MDH1 [*Candida glabrata*]	35.5	30.69	4.46	2	1	1	1	336	8.91
KAH7578664.1	MIR domain profile [*Candida glabrata*]	87.9	30.35	1.57	6	1	1	1	765	7.77
KAH7586162.1	Oxygen oxidoreductases covalent FAD-binding site [*Candida glabrata*]	59.7	30.17	2.29	2	1	1	1	525	7.50
SCV15969.1	related to Lysophospholipase 3 [*Candida glabrata*]	76.6	30.15	2.90	3	1	1	1	689	5.17
XP_446873.1	uncharacterized protein CAGL0H01705g [*Candida glabrata*]	14.8	29.47	4.41	1	1	1	1	136	8.62
KTB17879.1	Protein LDB19 [*Candida glabrata*]	87.8	29.43	0.75	4	1	1	1	795	7.43
KAH7591091.1	Isocitrate/isopropylmalate dehydrogenase [*Candida glabrata*]	46.8	29.12	1.70	7	1	1	1	411	5.36
KAH7583823.1	hypothetical protein [*Candida glabrata*]	14.4	28.99	4.24	19	1	1	1	118	9.66
XP_447581.1	uncharacterized protein CAGL0I07645g [*Candida glabrata*]	36.1	27.95	1.86	15	1	1	1	322	7.30

OXB44526.1	hypothetical protein [*Candida glabrata*]	115.0	27.91	0.70	10	1	1	1	1000	5.01
KAH7589082.1	hypothetical protein [*Candida glabrata*]	14.9	27.72	5.19	5	1	1	1	135	9.83
KAH7587748.1	G-protein alpha subunit [*Candida glabrata*]	51.0	27.33	2.47	3	1	1	1	446	7.66
XP_449470.1	40S ribosomal protein S0 [*Candida glabrata*]	27.9	27.08	3.19	1	1	1	1	251	4.83
QNG16511.1	uncharacterized protein CAGL0L08756g [*Candida glabrata*]	137.0	26.31	0.49	1	1	1	1	1219	6.32
SCV14749.1	Myosin-5 [*Candida glabrata*]	132.9	26.12	0.67	8	1	1	1	1191	9.42
SCV17043.1	probable Metal resistance protein YCF1 [*Candida glabrata*]	171.1	25.65	0.40	5	1	1	1	1514	8.24
KAH7590087.1	Jacalin-type lectin domain profile [*Candida glabrata*]	75.6	25.48	0.73	5	1	1	1	683	7.40
KAH7583940.1	PPR repeat family [*Candida glabrata*]	72.2	25.18	0.79	10	1	1	1	636	9.55
KAH7581023.1	Nudix box signature [*Candida glabrata*]	108.8	24.78	0.72	7	1	1	1	968	5.54
KAH7584919.1	hypothetical protein [*Candida glabrata*]	195.2	24.37	0.29	6	1	1	1	1710	5.47
KAH7587530.1	WSC domain [*Candida glabrata*]	42.3	22.64	5.76	9	1	1	1	399	5.48
SCV13136.1	probable Peptide transporter PTR2 [*Candida glabrata*]	65.7	21.99	1.20	5	1	1	1	581	5.88
KAH7579694.1	hypothetical protein [*Candida glabrata*]	118.3	21.46	0.56	4	1	1	1	1078	7.27
Candida parapsilosis										
XP_036663878.1	uncharacterized protein CPAR2_107330 [*Candida parapsilosis*]	37.4	432.84	22.44	1	5	5	8	352	5.24
XP_036668100.1	hypothetical protein CPAR2_407410 [*Candida parapsilosis*]	39.2	358.25	36.29	1	9	9	9	372	4.96
XP_036663998.1	hypothetical protein CPAR2_108560 [*Candida parapsilosis*]	44.4	342.23	19.86	1	7	7	7	433	4.46
XP_036664304.1	uncharacterized protein CPAR2_301150 [*Candida parapsilosis*]	31.7	334.56	28.72	1	5	5	7	296	9.60
KAF6048604.1	Glycosyl hydrolases 17 family protein [*Candida parapsilosis*]	55.9	294.17	8.53	3	5	5	6	551	4.59
XP_036665290.1	hypothetical protein CPAR2_806670 [*Candida parapsilosis*]	53.0	293.41	5.56	1	2	2	7	522	4.32
CAD1810552.1	unnamed protein product CANPARB_P26480 [*Candida parapsilosis*]	42.0	245.97	5.78	2	2	2	4	415	5.03
XP_036667234.1	uncharacterized protein CPAR2_502950 [*Candida parapsilosis*]	97.9	229.57	8.35	1	6	6	6	898	5.06
KAF6044433.1	hypothetical protein [*Candida parapsilosis*]	36.3	223.05	7.92	2	2	2	6	341	5.05
KAF6052363.1	SUR7/PalI family protein [*Candida parapsilosis*]	44.7	204.95	14.60	2	6	6	7	411	4.96
KAF6044400.1	ZIP Zinc transporter family protein [*Candida parapsilosis*]	67.9	156.94	5.02	3	2	2	2	618	5.00
XP_036666892.1	uncharacterized protein CPAR2_213690 [*Candida parapsilosis*]	57.5	149.56	3.20	1	1	1	1	532	4.48
XP_036666232.1	uncharacterized protein CPAR2_207060 [*Candida parapsilosis*]	50.0	142.59	14.63	5	6	6	6	458	8.95

XP_036664402.1	uncharacterized protein CPAR2_302140 [*Candida parapsilosis*]	59.3	142.30	6.58	1	3	3	3	532	4.58
CAD1811474.1	unnamed protein product CANPARB_P35700 [*Candida parapsilosis*]	28.3	124.42	13.60	3	3	3	3	272	4.25
XP_036667155.1	uncharacterized protein CPAR2_502160 [*Candida parapsilosis*]	49.6	120.34	6.26	1	2	2	2	463	4.39
XP_036663847.1	uncharacterized protein CPAR2_107020 [*Candida parapsilosis*]	11.5	120.22	21.36	1	2	2	3	103	11.36
XP_036667520.1	hypothetical protein CPAR2_401600 [*Candida parapsilosis*]	33.9	99.88	9.42	1	3	3	3	308	4.49
KAF6044595.1	Flocculin type 3 repeat family protein [*Candida parapsilosis*]	60.4	99.37	6.34	2	2	2	2	584	4.46
XP_036663157.1	uncharacterized protein CPAR2_100110 [*Candida parapsilosis*]	50.1	97.31	10.56	1	3	3	3	464	4.54
CAD1811016.1	unnamed protein product CANPARB_P31120 [*Candida parapsilosis*]	39.3	94.40	7.59	2	3	3	3	382	4.82
XP_036666900.1	uncharacterized protein CPAR2_213780 [*Candida parapsilosis*]	74.5	93.84	4.97	6	3	3	3	684	5.01
XP_036665822.1	uncharacterized protein CPAR2_202950 [*Candida parapsilosis*]	39.9	92.72	5.99	1	2	2	2	384	4.83
XP_036667949.1	uncharacterized protein CPAR2_405910 [*Candida parapsilosis*]	32.9	70.34	9.93	1	3	3	3	282	4.70
XP_036663991.1	uncharacterized protein CPAR2_108490 [*Candida parapsilosis*]	38.7	69.91	5.81	1	2	2	2	344	5.19
CAD1812452.1	unnamed protein product CANPARB_P45400 [*Candida parapsilosis*]	30.5	68.93	12.10	3	2	2	2	281	5.25
KAF6055424.1	Chitinase 3 [*Candida parapsilosis*]	51.7	66.61	4.52	2	1	1	1	487	4.40
XP_036665128.1	hypothetical protein CPAR2_805040 [*Candida parapsilosis*]	23.3	66.28	5.63	1	1	1	1	213	9.03
KAF6064068.1	glyceraldehyde-3-phosphate dehydrogenase, type I [*Candida parapsilosis*]	34.3	63.65	7.21	2	2	2	3	319	6.43
XP_036663472.1	uncharacterized protein CPAR2_103280 [*Candida parapsilosis*]	14.5	60.18	6.72	1	1	1	1	134	10.35
XP_036665295.1	uncharacterized protein CPAR2_806720 [*Candida parapsilosis*]	39.1	59.65	4.83	1	2	2	2	352	5.87
XP_036663678.1	uncharacterized protein CPAR2_105340 [*Candida parapsilosis*]	58.9	59.00	2.56	1	2	2	2	547	8.47
XP_036668466.1	uncharacterized protein CPAR2_703200 [*Candida parapsilosis*]	50.9	58.60	7.10	2	3	3	3	465	6.19
CAD1808323.1	unnamed protein product CANPARB_P04260 [*Candida parapsilosis*]	89.3	56.25	2.72	3	1	1	1	809	7.81
XP_036667304.1	uncharacterized protein CPAR2_503650 [*Candida parapsilosis*]	46.7	56.20	3.15	1	1	1	2	444	4.88
XP_036663852.1	uncharacterized protein CPAR2_107070 [*Candida parapsilosis*]	30.4	51.96	4.09	1	1	1	1	269	9.70
XP_036667400.1	uncharacterized protein CPAR2_400390 [*Candida parapsilosis*]	61.8	47.87	1.85	4	2	2	2	541	5.30
CAD1808766.1	unnamed protein product CANPARB_P08690 [*Candida parapsilosis*]	37.0	46.73	5.25	1	1	1	1	362	4.37
XP_036668337.1	uncharacterized protein CPAR2_701890 [*Candida parapsilosis*]	41.1	45.70	2.36	1	1	1	1	381	4.92
KAF6047521.1	hypothetical protein [*Candida parapsilosis*]	39.4	43.08	1.77	6	1	1	2	339	9.19
XP_036665638.1	uncharacterized protein CPAR2_201100 [*Candida parapsilosis*]	92.7	42.99	1.08	1	1	1	1	831	8.05

CAD1813253.1	unnamed protein product CANPARB_P53410 [*Candida parapsilosis*]	11.8	42.20	4.95	17	1	1	2	101	6.54
XP_036666226.1	uncharacterized protein CPAR2_207000 [*Candida parapsilosis*]	47.6	39.99	1.37	1	1	1	1	439	6.10
XP_036663512.1	uncharacterized protein CPAR2_103690 [*Candida parapsilosis*]	60.5	39.69	1.21	1	1	1	1	580	4.70
XP_036667059.1	uncharacterized protein CPAR2_501200 [*Candida parapsilosis*]	72.3	39.34	0.78	1	1	1	1	638	4.86
XP_036666709.1	uncharacterized protein CPAR2_211860 [*Candida parapsilosis*]	58.0	39.30	1.45	1	1	1	1	552	4.28
XP_036663337.1	uncharacterized protein CPAR2_101920 [*Candida parapsilosis*]	26.6	38.90	2.85	1	1	1	1	246	5.24
XP_036664704.1	uncharacterized protein CPAR2_800810 [*Candida parapsilosis*]	51.6	38.78	3.02	1	1	1	1	463	4.77
XP_036667190.1	uncharacterized protein CPAR2_502510 [*Candida parapsilosis*]	32.5	38.59	1.81	15	1	1	1	277	5.86
XP_036664472.1	uncharacterized protein CPAR2_302840 [*Candida parapsilosis*]	41.4	37.82	2.25	1	1	1	2	356	6.48
XP_036663694.1	uncharacterized protein CPAR2_105500 [*Candida parapsilosis*]	33.9	36.49	3.29	2	1	1	1	304	4.92
XP_036667977.1	uncharacterized protein CPAR2_406190 [*Candida parapsilosis*]	77.6	36.28	0.89	1	1	1	1	676	5.24
XP_036663854.1	uncharacterized protein CPAR2_107090 [*Candida parapsilosis*]	14.1	35.83	6.87	1	1	1	2	131	10.14
XP_036667811.1	uncharacterized protein CPAR2_404530 [*Candida parapsilosis*]	27.8	35.63	3.57	1	1	1	1	252	5.27
XP_036667283.1	uncharacterized protein CPAR2_503450 [*Candida parapsilosis*]	43.7	34.59	1.81	1	1	1	1	387	11.08
CAD1808736.1	unnamed protein product CANPARB_P08390 [*Candida parapsilosis*]	35.4	32.80	4.29	3	1	1	1	326	4.88
CAD1807947.1	unnamed protein product CANPARB_P00500 [*Candida parapsilosis*]	37.3	32.78	3.08	2	1	1	1	325	6.40
XP_036666469.1	uncharacterized protein CPAR2_209470 [*Candida parapsilosis*]	50.0	32.41	1.12	1	1	1	1	448	6.42
KAF6042178.1	Erv1 / Alr family protein [*Candida parapsilosis*]	28.2	31.65	3.98	2	1	1	1	251	6.67
KAF6048200.1	Eukaryotic aspartyl protease family protein [*Candida parapsilosis*]	63.9	31.56	2.01	2	1	1	1	597	4.81
XP_036666914.1	uncharacterized protein CPAR2_213920 [*Candida parapsilosis*]	59.1	29.89	2.07	1	1	1	1	532	7.96
XP_036665197.1	uncharacterized protein CPAR2_805730 [*Candida parapsilosis*]	19.6	29.33	6.21	1	1	1	1	177	6.54
XP_036666891.1	uncharacterized protein CPAR2_213680 [*Candida parapsilosis*]	25.6	29.22	2.65	1	1	1	1	226	9.33
XP_036668566.1	uncharacterized protein CPAR2_704210 [*Candida parapsilosis*]	138.2	28.54	0.52	1	1	1	1	1164	9.48
CAD1809699.1	unnamed protein product CANPARB_P18020 [*Candida parapsilosis*]	217.4	28.48	0.36	2	1	1	1	1954	6.25
XP_036667769.1	uncharacterized protein CPAR2_404100 [*Candida parapsilosis*]	60.2	28.44	0.92	3	1	1	1	543	6.80
KAF6048267.1	hypothetical protein [*Candida parapsilosis*]	11.7	28.27	4.90	6	1	1	1	102	5.29
CAD1813697.1	unnamed protein product CANPARB_P57850 [*Candida parapsilosis*]	46.4	28.20	1.18	1	1	1	1	422	5.62
XP_036664728.1	uncharacterized protein CPAR2_801050 [*Candida parapsilosis*]	37.1	27.70	1.42	5	1	1	1	353	4.78

XP_036667906.1	uncharacterized protein CPAR2_405480 [*Candida parapsilosis*]	110.6	26.94	0.52	1	1	1	1	960	6.87
CAD1808677.1	unnamed protein product CANPARB_P07800 [*Candida parapsilosis*]	48.9	26.93	1.39	2	1	1	1	433	5.99
XP_036665399.1	uncharacterized protein CPAR2_807760 [*Candida parapsilosis*]	23.8	26.65	2.88	2	1	1	1	208	4.82
KAF6048353.1	Methyltransferase domain family protein [*Candida parapsilosis*]	26.0	26.22	3.90	2	1	1	1	231	4.78
XP_036666773.1	uncharacterized protein CPAR2_212500 [*Candida parapsilosis*]	40.6	26.18	1.99	1	1	1	1	352	6.70
XP_036663055.1	uncharacterized protein CPAR2_602890 [*Candida parapsilosis*]	60.5	26.01	1.55	1	1	1	1	517	8.85
XP_036662990.1	uncharacterized protein CPAR2_602230 [*Candida parapsilosis*]	185.6	25.78	0.37	1	1	1	1	1635	5.25
XP_036664238.1	uncharacterized protein CPAR2_300490 [*Candida parapsilosis*]	191.0	25.63	0.78	1	1	1	1	1670	5.94
KAF6046219.1	Surface antigen protein 2 [*Candida parapsilosis*]	82.0	25.47	1.25	8	1	1	1	798	3.92
XP_036665988.1	uncharacterized protein CPAR2_204610 [*Candida parapsilosis*]	31.1	25.10	1.80	1	1	1	1	278	5.72
CAD1809173.1	unnamed protein product CANPARB_P12760 [*Candida parapsilosis*]	81.2	24.15	0.70	5	1	1	1	716	8.75
ABE27299.1	mitochondrial F-ATPase beta subunit, partial [*Candida parapsilosis*]	35.9	24.04	3.26	2	1	1	1	337	5.05
XP_036667847.1	uncharacterized protein CPAR2_404890 [*Candida parapsilosis*]	17.7	23.88	5.45	1	1	1	1	165	9.42
XP_036666189.1	uncharacterized protein CPAR2_206630 [*Candida parapsilosis*]	40.6	23.56	1.62	1	1	1	1	371	6.80
XP_036668062.1	uncharacterized protein CPAR2_407030 [*Candida parapsilosis*]	64.8	23.39	1.04	2	1	1	1	577	8.37
KAF6045709.1	ABC transporter transmembrane region family protein [*Candida parapsilosis*]	162.9	23.11	0.41	3	1	1	1	1447	5.69
XP_036664853.1	uncharacterized protein CPAR2_802280 [*Candida parapsilosis*]	73.8	22.07	1.09	1	1	1	1	645	5.06
Candida tropicalis										
XP_002549456.1	pH-regulated antigen PRA1 precursor [*Candida tropicalis* MYA-3404]	34.8	837.95	30.89	1	9	9	26	314	4.55
XP_002549000.1	glucoamylase 1 precursor [*Candida tropicalis* MYA-3404]	102.5	414.29	17.27	1	14	14	16	915	4.81
XP_002546120.1	hypothetical protein CTRG_00902 [*Candida tropicalis* MYA-3404]	52.5	303.05	11.78	1	1	4	4	467	4.51
KAG4405589.1	hypothetical protein [*Candida tropicalis*]	44.0	258.44	19.26	1	7	7	7	431	4.53
XP_002546108.1	hypothetical protein CTRG_00890 [*Candida tropicalis* MYA-3404]	52.5	249.20	12.04	1	1	4	4	465	4.58
XP_002547786.1	hypothetical protein CTRG_02093 [*Candida tropicalis* MYA-3404]	38.8	244.43	21.35	1	5	5	6	370	4.93
XP_002549039.1	hypothetical protein [*Candida tropicalis* MYA-3404]	55.2	225.77	8.55	5	4	4	5	503	4.70
XP_002549896.1	hypothetical protein CTRG_04193 [*Candida tropicalis* MYA-3404]	120.4	190.82	4.52	1	4	4	5	1083	4.74
XP_002545265.1	predicted protein CTRG_00046 [*Candida tropicalis* MYA-3404]	25.2	180.65	18.44	1	3	3	3	244	4.41
XP_002549841.1	conserved hypothetical protein CTRG_04138 [*Candida tropicalis* MYA-3404]	21.4	178.28	13.13	2	2	2	3	198	4.22

XP_002547549.1	predicted protein CTRG_01856 [*Candida tropicalis* MYA-3404]	65.5	178.12	5.07	1	3	3	6	631	4.12
XP_002546355.1	conserved hypothetical protein CTRG_05833 [*Candida tropicalis* MYA-3404]	37.9	162.36	16.57	1	5	5	5	332	5.97
XP_002549562.1	conserved hypothetical protein CTRG_03859 [*Candida tropicalis* MYA-3404]	37.5	150.38	13.56	1	4	4	5	354	5.00
XP_002546924.1	hypothetical protein CTRG_01230 [*Candida tropicalis* MYA-3404]	74.8	132.91	3.98	1	2	2	2	679	4.56
KAG4407032.1	hypothetical protein [*Candida tropicalis*]	41.8	131.07	11.17	6	5	5	5	376	5.48
XP_002546360.1	predicted protein CTRG_05838 [*Candida tropicalis* MYA-3404]	194.6	125.49	3.69	2	3	3	5	1952	3.74
XP_002551160.1	hypothetical protein CTRG_05458 [*Candida tropicalis* MYA-3404]	58.6	120.46	6.67	1	3	3	3	540	4.09
XP_002550650.1	hypothetical protein CTRG_04948 [*Candida tropicalis* MYA-3404]	181.8	118.09	1.56	2	3	3	3	1797	3.89
XP_002548866.1	enolase 1 [*Candida tropicalis* MYA-3404]	47.0	98.05	6.59	1	2	2	2	440	5.85
XP_002546966.1	predicted protein CTRG_01272 [*Candida tropicalis* MYA-3404]	163.6	94.07	2.03	1	3	3	3	1628	3.87
XP_002547548.1	predicted protein CTRG_01855 [*Candida tropicalis* MYA-3404]	108.6	93.40	2.82	1	3	3	3	1065	4.11
XP_002545388.1	glucan 1,3-beta-glucosidase precursor [*Candida tropicalis* MYA-3404]	34.0	92.22	14.24	1	4	4	4	309	4.46
XP_002546635.1	alcohol dehydrogenase 1 [*Candida tropicalis* MYA-3404]	43.5	84.45	7.35	2	2	3	3	408	8.22
XP_002547526.1	elongation factor 1-alpha [*Candida tropicalis* MYA-3404]	49.9	83.80	4.37	6	2	2	2	458	9.03
XP_002548401.1	predicted protein CTRG_02698 [*Candida tropicalis* MYA-3404]	46.2	78.16	5.19	4	2	2	3	424	4.39
XP_002545510.1	predicted protein CTRG_00291 [*Candida tropicalis* MYA-3404]	64.8	77.58	3.20	4	2	2	2	656	3.66
XP_002551368.1	glyceraldehyde-3-phosphate dehydrogenase [*Candida tropicalis* MYA-3404]	36.0	76.06	10.42	3	3	3	3	336	6.65
XP_002551158.1	chitinase 2 precursor [*Candida tropicalis* MYA-3404]	68.8	70.03	3.23	1	2	2	2	651	4.27
XP_002551325.1	ubiquitin [*Candida tropicalis* MYA-3404]	8.7	64.13	19.48	3	2	2	3	77	7.25
XP_002549044.1	predicted protein CTRG_03341 [*Candida tropicalis* MYA-3404]	22.8	58.44	5.19	1	1	1	1	212	5.06
XP_002549287.1	opaque-phase-specific protein OP4 precursor [*Candida tropicalis* MYA-3404]	43.3	57.38	4.04	1	1	1	1	421	5.06
XP_002546246.1	conserved hypothetical protein CTRG_01028 [*Candida tropicalis* MYA-3404]	79.0	56.67	2.17	2	1	1	1	738	4.60
XP_002550610.1	predicted protein CTRG_04908 [*Candida tropicalis* MYA-3404]	19.7	55.06	13.48	1	1	1	2	178	4.89
ACX81423.1	alcohol dehydrogenase, partial [*Candida tropicalis*]	33.2	54.84	6.96	2	1	2	2	316	7.65
XP_002549645.1	protein EPD2 precursor [*Candida tropicalis* MYA-3404]	60.5	54.47	5.07	1	2	2	2	552	4.49
XP_002545666.1	predicted protein CTRG_00447 [*Candida tropicalis* MYA-3404]	40.8	54.39	2.99	1	1	1	1	368	4.42
QDZ60013.1	agglutinin-like adhesin protein [*Candida tropicalis*]	101.7	51.23	1.13	2	1	1	1	974	3.90
XP_002550430.1	heat shock protein 70 2 [*Candida tropicalis* MYA-3404]	70.0	50.27	1.71	3	1	1	1	644	5.06

XP_002545651.1	hypothetical protein CTRG_00432 [*Candida tropicalis* MYA-3404]	36.8	49.39	3.70	1	1	1	1	324	4.74
XP_002545668.1	histone H2A variant [*Candida tropicalis* MYA-3404]	21.5	47.90	4.66	1	1	1	1	193	10.39
XP_002546878.1	conserved hypothetical protein CTRG_01183 [*Candida tropicalis* MYA-3404]	65.9	47.55	2.25	1	1	1	1	623	4.42
XP_002548327.1	histone H2B.1 [*Candida tropicalis* MYA-3404]	14.1	47.47	6.92	1	1	1	2	130	10.10
XP_002545482.1	hypothetical protein CTRG_00263 [*Candida tropicalis* MYA-3404]	49.6	47.12	2.33	1	1	1	1	473	4.46
AAF63792.1	heat shock protein 90, partial [*Candida tropicalis*]	78.9	46.84	0.87	1	1	1	2	690	4.89
XP_002547953.1	guanosine-diphosphatase [*Candida tropicalis* MYA-3404]	64.1	43.87	1.20	1	1	1	1	582	5.38
XP_002550778.1	FAD-linked sulfhydryl oxidase ERV2 [*Candida tropicalis* MYA-3404]	26.4	41.32	4.33	1	1	1	1	231	5.87
XP_002547857.1	protein TOS1 precursor [*Candida tropicalis* MYA-3404]	49.8	40.83	3.01	1	1	1	2	465	4.70
XP_002547980.1	predicted protein CTRG_02277 [*Candida tropicalis* MYA-3404]	22.1	40.60	2.67	10	1	1	2	187	8.25
XP_002546142.1	hypothetical protein CTRG_00924 [*Candida tropicalis* MYA-3404]	99.6	38.70	1.15	9	1	1	2	866	8.38
XP_002547660.1	predicted protein CTRG_01967 [*Candida tropicalis* MYA-3404]	78.6	38.47	1.63	1	1	1	1	734	3.83
XP_002546324.1	predicted protein CTRG_05802 [*Candida tropicalis* MYA-3404]	57.8	36.19	1.17	1	1	1	1	514	6.24
XP_002549878.1	conserved hypothetical protein CTRG_04175 [*Candida tropicalis* MYA-3404]	14.5	35.56	4.80	1	1	1	1	125	9.09
XP_002545947.1	predicted protein CTRG_00728 [*Candida tropicalis* MYA-3404]	138.9	35.56	0.55	1	1	1	1	1267	4.60
XP_002550134.1	conserved hypothetical protein CTRG_04432 [*Candida tropicalis* MYA-3404]	36.7	35.37	2.15	1	1	1	1	325	4.94
XP_002551076.1	conserved hypothetical protein CTRG_05374 [*Candida tropicalis* MYA-3404]	58.8	35.21	3.24	1	2	2	2	525	10.40
XP_002550400.1	ATP synthase alpha chain, mitochondrial precursor [*Candida tropicalis* MYA-3404]	48.5	34.79	1.57	2	1	1	1	447	8.50
XP_002549807.1	hypothetical protein CTRG_04104 [*Candida tropicalis* MYA-3404]	153.0	34.76	0.44	2	1	1	1	1363	9.31
XP_002548231.1	hypothetical protein CTRG_02528 [*Candida tropicalis* MYA-3404]	106.7	33.27	0.54	1	1	1	2	918	7.11
YP_008474987.1	putative LAGLIDADG endonuclease, partial [*Candida tropicalis*]	40.6	31.60	1.46	1	1	1	1	343	9.26
XP_002550720.1	peptidyl-prolyl cis-trans isomerase [*Candida tropicalis* MYA-3404]	14.2	31.58	6.25	1	1	1	1	128	9.14
XP_002546667.1	glycolipid-anchored surface protein 5 precursor [*Candida tropicalis* MYA-3404]	50.1	31.49	2.39	1	1	1	1	460	4.41
XP_002551378.1	predicted protein CTRG_05676 [*Candida tropicalis* MYA-3404]	74.9	30.68	1.09	1	1	1	1	645	4.22
AAF81927.1	elongation factor 2, partial [*Candida tropicalis*]	89.7	30.38	0.98	3	1	1	1	813	6.89
XP_002545304.1	conserved hypothetical protein CTRG_00085 [*Candida tropicalis* MYA-3404]	46.9	30.17	1.21	1	1	1	1	412	5.47
XP_002548057.1	conserved hypothetical protein CTRG_02354 [*Candida tropicalis* MYA-3404]	82.2	29.77	0.67	1	1	1	1	748	6.39
XP_002545238.1	hypothetical protein CTRG_00019 [*Candida tropicalis* MYA-3404]	29.6	29.62	2.71	1	1	1	1	258	6.92

XP_002550193.1	candidapepsin-7 precursor [*Candida tropicalis* MYA-3404]	73.0	29.46	1.16	1	1	1	1	692	4.60
XP_002548571.1	predicted protein CTRG_02868 [*Candida tropicalis* MYA-3404]	56.0	29.35	1.23	1	1	1	1	486	5.06
XP_002547543.1	conserved hypothetical protein CTRG_01850 [*Candida tropicalis* MYA-3404]	87.7	28.77	0.79	1	1	1	1	764	5.59
XP_002550478.1	predicted protein CTRG_04776 [*Candida tropicalis* MYA-3404]	48.8	27.32	1.86	1	1	1	1	431	6.00
XP_002547151.1	conserved hypothetical protein CTRG_01457 [*Candida tropicalis* MYA-3404]	40.7	26.19	1.98	1	1	1	1	353	5.20
XP_002550020.1	hypothetical protein CTRG_04317 [*Candida tropicalis* MYA-3404]	63.5	25.86	1.07	1	1	1	1	561	6.07
XP_002548247.1	predicted protein CTRG_02544 [*Candida tropicalis* MYA-3404]	137.6	25.45	0.42	1	1	1	1	1193	6.73
XP_002547546.1	structural maintenance of chromosome 2 [*Candida tropicalis* MYA-3404]	133.1	25.13	0.94	1	1	1	1	1171	7.01
XP_002545782.1	predicted protein CTRG_00563 [*Candida tropicalis* MYA-3404]	32.4	24.52	2.12	1	1	1	1	283	8.82
XP_002549280.1	predicted protein CTRG_03577 [*Candida tropicalis* MYA-3404]	38.2	24.37	1.81	1	1	1	1	332	6.29
XP_002551086.1	conserved hypothetical protein CTRG_05384 [*Candida tropicalis* MYA-3404]	25.5	24.30	2.35	1	1	1	1	213	8.85
XP_002549371.1	protein NCA3, mitochondrial precursor [*Candida tropicalis* MYA-3404]	39.4	24.27	3.20	1	1	1	1	375	4.87
XP_002545929.1	protein kinase byr1 [*Candida tropicalis* MYA-3404]	61.9	23.91	1.08	1	1	1	1	557	8.31
XP_002548095.1	60S ribosomal protein L12 [*Candida tropicalis* MYA-3404]	17.7	23.38	5.45	1	1	1	1	165	9.51
P53031.1	Protein phosphatase PP2A regulatory subunit B [*Candida tropicalis*]	57.9	22.89	1.57	2	1	1	1	508	5.05
XP_002550607.1	predicted protein CTRG_04905 [*Candida tropicalis* MYA-3404]	19.5	22.34	5.71	1	1	1	1	175	4.54
XP_002549668.1	hypothetical protein CTRG_03965 [*Candida tropicalis* MYA-3404]	65.6	22.13	1.51	1	1	1	1	598	5.77
KAG4407464.1	hypothetical protein [*Candida tropicalis*]	31.7	20.94	2.89	1	1	1	1	277	4.56
XP_002548472.1	hypothetical protein CTRG_02769 [*Candida tropicalis* MYA-3404]	103.0	20.38	0.67	1	1	1	1	890	6.13
XP_002548607.1	conserved hypothetical protein CTRG_02904 [*Candida tropicalis* MYA-3404]	18.8	20.37	6.32	1	1	1	1	174	4.68
